# Portable NIR Spectroscopy to Simultaneously Trace Honey Botanical and Geographical Origins and Detect Syrup Adulteration

**DOI:** 10.3390/foods13193062

**Published:** 2024-09-26

**Authors:** Marco Caredda, Marco Ciulu, Francesca Tilocca, Ilaria Langasco, Oscar Núñez, Sònia Sentellas, Javier Saurina, Maria Itria Pilo, Nadia Spano, Gavino Sanna, Andrea Mara

**Affiliations:** 1 Department of Animal Science, AGRIS Sardegna, Loc. Bonassai, 07100 Sassari, Italy; mcaredda@agrisricerca.it; 2 Department of Biotechnology, University of Verona, Strada le Grazie 15, 37134 Verona, Italy; marco.ciulu@univr.it; 3 Department of Chemical, Physical, Mathematical, and Natural Sciences, University of Sassari, Via Vienna 2, 07100 Sassari, Italy; fra.til@hotmail.com (F.T.); ilangasco@uniss.it (I.L.); mpilo@uniss.it (M.I.P.); nspano@uniss.it (N.S.); sanna@uniss.it (G.S.); 4 Department of Chemical Engineering and Analytical Chemistry, University of Barcelona, Martí i Franquès 1-11, 08028 Barcelona, Spain; oscar.nunez@ub.edu (O.N.); sonia.sentellas@ub.edu (S.S.); xavi.saurina@ub.edu (J.S.); 5 Research Institute in Food Nutrition and Food Safety, University of Barcelona, Recinte Torribera, Av. Prat de la Riba 171, Edifici de Recerca (Gaudí), Santa Coloma de Gramenet, 08921 Barcelona, Spain; 6 Departament de Recerca I Universitats, Generalitat de Catalunya, Via Laietana 2, 08003 Barcelona, Spain

**Keywords:** honey, near-infrared spectroscopy, adulteration, geographical origin, botanical origin

## Abstract

Fraudulent practices concerning honey are growing fast and involve misrepresentation of origin and adulteration. Simple and feasible methods for honey authentication are needed to ascertain honey compliance and quality. Working on a robust dataset and simultaneously investigating honey traceability and adulterant detection, this study proposed a portable FTNIR fingerprinting approach combined with chemometrics. Multifloral and unifloral honey samples (*n* = 244) from Spain and Sardinia (Italy) were discriminated by botanical and geographical origin. Qualitative and quantitative methods were developed using linear discriminant analysis (LDA) and partial least squares (PLS) regression to detect adulterated honey with two syrups, consisting of glucose, fructose, and maltose. Botanical and geographical origins were predicted with 90% and 95% accuracy, respectively. LDA models discriminated pure and adulterated honey samples with an accuracy of over 92%, whereas PLS allows for the accurate quantification of over 10% of adulterants in unifloral and 20% in multifloral honey.

## 1. Introduction

Beekeeping is facing significant challenges in the agri-food sector due to climate change [1], the excessive use of agrochemicals [2], the spread of the Varroa parasite [3], industrialization [4], and the presence of adulterated and counterfeit products on the market sold at low prices [5]. This last aspect has recently been highlighted by a coordinated action led by the European Commission’s Directorate-General for Health and Food Safety (DG SANTE), along with the national authorities of 18 countries, the European Anti-Fraud Office (OLAF), and the European Commission’s Joint Research Centre (JRC). This action revealed several fraudulent practices, highlighting the need for new analytical techniques to verify the authenticity of bee products [6].

Considering recent survey data [6], a reliable analytical approach is required for detecting adulterants in honey and tracing its origin. The methods used for routine control should be accurate and suitable. For instance, they may be useful in monitoring honey imports from non-EU countries and the internal market, which are both susceptible to fraud and adulteration. In this framework, the capacity to conduct on-site analysis using portable instruments is desirable for food supply chain control.

Authenticity is a crucial aspect of honey quality. It also refers to compliance with declared information, quality regulations, and safety [7,8]. According to European legislation, the honey label must indicate whether the honey comes from the EU, non-EU countries, or a mixture of both. Additional information, such as floral and territorial attribution, may also be included [9] as these features determine the organoleptic properties of honey [10,11]. Indeed, consumers are becoming increasingly aware of the characteristics of honey and are making purchasing decisions based on its stated floral or territorial origin [12,13]. In addition to honey quality standards, European legislation states that honey must not contain adulterants. The most commonly used are syrups from C3 and C4 plants, such as corn syrup and rice syrup [14,15], or glucose and sucrose syrups derived from beets or canes [16].

Analytical methods for food authentication can be classified according to the approach employed [17]. The targeted approach focuses on the detection and quantification of specific known compounds or markers, which are characteristic of a particular food class, to verify its authenticity. The untargeted approach, which is also referred to as fingerprinting, involves a comprehensive analysis without prior knowledge of specific compounds. This approach aims to identify and profile all detectable substances to discover patterns or markers that are indicative of authenticity.

Several analytical techniques can be used to authenticate honey using both approaches, including elemental analysis [18,19], isotope pattern determination [20,21], electronic sensors [22,23], and chromatography or hyphenated methodologies [24,25,26,27]. Similarly, spectroscopic methods such as NMR [28], Raman spectroscopy [29,30], spectrofluorimetry [31], UV-Vis spectroscopy [25,32], and infrared (IR) or near-infrared (NIR) spectroscopy [24,33,34] have also been used for this purpose, offering several advantages. For instance, although mass spectrometry-based methods guarantee excellent analytical performance in terms of sensitivity and versatility, they typically require sample preparation procedures, including solid-phase and liquid–liquid extraction or even energy-assisted methods (such as microwave-assisted and pressurized-liquid extraction) [24]. Conversely, spectroscopic analysis frequently necessitates a reduced number of sample preparation steps or enables noninvasive measurement [35]. Furthermore, the time required for analysis is typically longer for chromatographic techniques, with a single run often lasting several minutes [36]. In comparison, spectroscopic methods can rapidly obtain a spectrum or output, which can be directly used for quality control or detect adulteration. For this reason, spectroscopic methods are particularly well suited to use with an untargeted approach, especially when combined with machine learning and/or chemometric techniques that maximize information in complex and large datasets [37,38,39]. Additionally, spectroscopic techniques are more straightforward, and the instrumentation is more cost-effective and user-friendly. In conclusion, considering these aspects, an untargeted spectroscopic approach satisfies all the requirements needed for a screening method that aims to preliminarily analyze suspected counterfeit honeys.

Among the spectroscopic methods, IR and NIR are among the most frequently used as they can detect thermal treatments [40] and adulteration [15,16,41,42,43], classify honey according to botanical and/or geographical origin [33,39,44,45,46,47], and predict chemical and physical properties [48]. For example, the NIR region is useful for determining honey authenticity because major sugar variations influence it [49,50,51,52]. Both analytical techniques have numerous advantages, and IR spectroscopy, especially when coupled with an ATR (attenuated total reflectance) device, allows for fast and simple analysis. However, NIR spectroscopy is also a consolidated analytical method in the food industry that allows for the prediction of food composition and functional properties [53]. It also enables food safety evaluation and quality control, guaranteeing easy and fast analysis, noninvasive measurement, or minimal sample preparation [35]. Moreover, the recent portable NIR instrumentation allows for obtaining great optical performance and is equipped with detectors similar to those present in benchtop devices [54].

Recently, portable NIR spectrophotometers have been tested for honey quality control [55] and fraud detection [49,56]. Escuredo et al. [55] reported that moisture, hydroxymethylfurfural, color, and flavonoids can be accurately predicted. Guelpa et al. [49] distinguished authentic South African honey from imported or adulterated samples. Nevertheless, the authors did not differentiate samples according to their botanical origin, and the geographical discrimination is not well supported by the limited sampling, as well as the adulteration study. Folli et al. [56] tested the detection of honey adulterated with nectar, glucose, and sugarcane molasses. However, even in this case, the study analyzed only five commercial and uncharacterized samples. Therefore, although some preliminary findings have been previously presented, the realistic feasibility of the approach has yet to be demonstrated. A larger dataset is required to consider the high variability in honey saccharide composition, both in terms of botanical and geographical origin. Similarly, the variability in sugar composition should be considered in adulteration studies, which require larger datasets for model calibration. In conclusion, although portable NIR spectroscopy shows great potential, further testing is required to demonstrate its effectiveness as a screening method for predicting botanical and geographical origins and in detecting adulterations.

This study aimed therefore to evaluate NIR portable spectroscopy as a screening approach for combating honey fraud by tracing botanical and geographical origin and detecting adulterants. Given the lack of robustness of previously reported work, this study has been conducted by analyzing a large dataset composed of both unifloral and multifloral honey from different geographical origins. Furthermore, the influence of botanical origins on adulterant detection has been investigated since previous studies never considered both aspects simultaneously. To propose a fast and simple method, the data processing included simple chemometric tools that are commonly implemented in instrument software. Therefore, advanced or machine learning techniques were not considered at this stage. A total of 244 multifloral and unifloral honey samples from Spain and Sardinia (Italy) were analyzed. These countries were compared because they share similar floral sources and pedology [18]. As previously reported, these conditions increase the difficulties in honey traceability studies; however, they ensure that the geographical discrimination is less significantly influenced by the comparison of different botanical origins. In addition, 720 adulterated samples were prepared using two adulterant syrups with different saccharide compositions. Linear discriminant analysis (LDA) was used to discriminate samples based on botanical and geographic origin, while partial least squares (PLS) regression was used for adulterant quantification. Different spectral pretreatments were evaluated while genetic algorithms (GAs) were used for variable selection [57]. The GA variable selection offers a very good solution in terms of both predictive ability and interpretability and does not require any spectroscopic experience by the user [58]. A particular feature of GAs is that they individuate not only the expected bands but also additional, sometimes unexpected, bands whose presence allows for an increase in the predictivity of the model [59].

## 2. Materials and Methods

### 2.1. Honey Samples and Adulterants

This study analyzed 244 honey samples from Spain (*n* = 71) and Sardinia (Italy, *n* = 173) collected between 2020 and 2022, according to the flowering and seasonality of the botanical sources [18]. The collection included both unifloral and multifloral honey. It consists of 68 multifloral honeys (35 from Sardinia and 33 from Spain), 42 eucalyptus honeys (30 from Sardinia and 12 from Spain), 32 rosemary honeys (6 from Sardinia and 26 from Spain) and 3 varieties of typical Sardinian unifloral honeys, 37 thistle honeys, 36 asphodel honeys, and 29 strawberry tree honeys [60]. These botanical varieties were selected based on common botanical sources between the two geographical areas. In addition, some peculiar unifloral honeys of Sardinia were taken into account to compare also uncommon botanical origins [60]. The melissopalynological analysis is reported in Table S1. All the samples were stored in the dark at 4 °C until analysis.

Two syrups were prepared and tested as adulterants. The first (AD1) was a colorless syrup composed of glucose (26%), maltose (32%), fructose (17%), and water (25%). The second (AD2) was a pale yellow syrup composed of glucose (19%), fructose (56%), and water (25%). The composition of the adulterants was chosen based on the composition of honey and the most commonly used adulterants, such as rice and corn syrup [36]. Honey contains mainly fructose (35–40%) and glucose (30-35%) in varying proportions depending on the botanical and geographical origin [61]. Other sugars in honey, such as maltose, are present in concentrations less than 10%. Adulterant syrups may contain several carbohydrates in different percentages. Corn syrup, and in particular high fructose corn syrup (HFCS), is usually employed for honey adulteration as it predominantly contains glucose and fructose in high percentages. On the other hand, rice syrup can contain high percentages of maltose and other higher sugars that are derived from the hydrolysis of starch [62,63]. Therefore, the syrups used in this study are composed of fructose, glucose, and maltose to evaluate the detection of the commercial adulterants most used for honey counterfeiting.

### 2.2. Reagents and Instrumentations

Saccharides, D-(+)-Glucose (BioXtra, ≥99% (GC)), D-(+)-Maltose monohydrate (BioXtra, ≥99%), and D-(−)-Fructose (BioXtra, ≥99%), were from Sigma Aldrich (St. Louis, MI, USA). Type I water (resistivity > 18 MΩ cm^−1^) was produced using a MilliQ Plus System from Millipore (Milan, Italy) and used in all the analytical phases. The samples were homogenized using an Ultra-Turrax mixer mod T18 (IKA, Staufen, Germany). The FT-NIR spectra of honey were acquired using a miniaturized MicroNIR OnSite-W (VIAVI, Santa Rosa, CA, USA) equipped with a tungsten light source, linear-variable filter connected to an InGaAs array detector for NIR measurements and a vial holder for liquid analysis [54,64].

### 2.3. Sample Preparation and Spectra Acquisition

All the samples were homogenized and heated in a thermostatic water bath at 35 °C. The spectra were acquired in triplicate at 25 °C, ranging from 908 to 1676 nm with 100 scans and 6.15 nm resolution, resulting in spectra characterized by 125 wavelengths. The samples were analyzed in duplicate. The reference spectra were collected every 10 min. The assignment of specific spectral regions to the corresponding absorption of the chemical functional group was performed according to the literature [65,66,67,68,69]. The spectral analysis is discussed in Section S1 of the Supplementary Material. Figure S1 shows the honey spectra colored according to botanical origin. 

### 2.4. Chemometric Analysis

**Figure 1:**
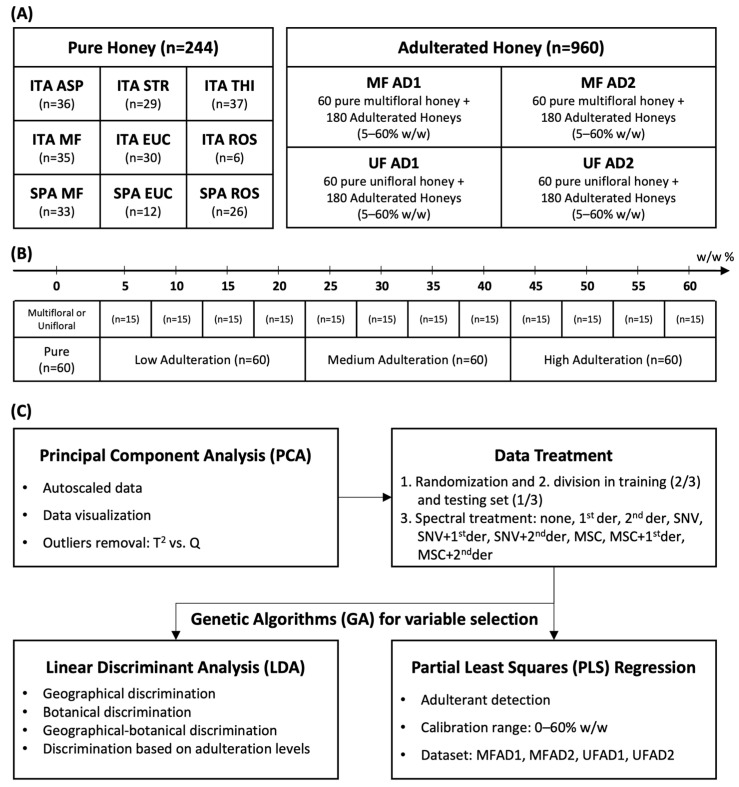
(**A**) Composition of datasets and geographical–botanical origins of honeys. (**B**) Adulteration levels and categories. (**C**) Workflow of chemometric analysis. ITA = Sardinia (Italy); SPA = Spain; ASP = asphodel; STR = strawberry tree; THI = thistle; MF = multifloral; EUC = eucalyptus; ROS = rosemary; AD1 = adulterant syrup of glucose–maltose–fructose (26%–33%–17%, moisture 25%); AD2 = adulterant syrup of glucose and fructose (19%–56%, moisture 25%); 1st der = first derivate; 2nd der = second derivate; SNV = Standard Normal Variate; and MSC = multiplicative scatter correction.

The workflow of the chemometric analysis is summarized in Figure 1. The pure honey samples (244) were randomly analyzed to acquire the FT-NIR spectra, which were used to develop classification models for the traceability of the geographical and botanical origin. The unifloral honeys (15 asphodel, 15 eucalyptus, 15 strawberry tree, and 15 thistle) and multifloral honeys (30 Spanish and 30 Sardinian) were adulterated with syrups AD1 and AD2, respectively.

The statistical models for the detection of adulterants were calculated separately, distinguishing between the unifloral and multifloral honey samples (Tables S2 and S3). Each dataset consisted of 240 samples, 60 pure and 180 adulterated, with low (5, 10, 15, and 20%), medium (25, 30, 35, and 40%), and high (45, 50, 55, and 60%) concentrations of syrup. For each dataset (Figure 1A), a principal component analysis (PCA) of the NIR spectra was run to visualize the data and detect possible outliers using T^2^ vs. Q diagnostics. A linear discriminant analysis (LDA) was used to build discriminant models. Genetic algorithms (GAs) were employed to reduce the spectral variables [58]. This was necessary because LDA cannot be performed when there are numerous and highly correlated variables. Predictive models were built using partial least squares (PLS) regression with both full-spectra and GA variable selection. The datasets were randomly divided into a training set (two-thirds of the total samples) and a test set (one-third of the total samples) for external validation. The training set was used to calculate the models performing a 5-fold cross-validation. For each model, the raw spectra, Standard Normal Variate (SNV)-transformed spectra, and multiplicative scatter correction (MSC)-transformed spectra were evaluated. Each pretreatment was evaluated by applying either first- or second-derivative functions, resulting in nine different combinations of data pretreatment methods (Figure 1B). The honey samples were discriminated according to their botanical and geographical origins using LDA (Figure 1C). The datasets used for LDA modeling consisted of equally partitioned categories. The results obtained in the LDA confusion matrices were evaluated in terms of accuracy and calculated as the percentage average of correct predictions of each category. Additionally, an LDA was run to discriminate four categories at different levels of adulteration (Figure 1B). The quantification models were created using PLS regression (Figure 1C). PCA was run on Minitab 16.2.0 on autoscaled data, whereas PLS and LDA were performed using the Chemometric Agile Tool (CAT) software (R version 3.1.2) [70]. Finally, the GAs were run on MATLAB (R2021a release) with PLS-GA Toolbox [58].

## 3. Results and Discussion

### 3.1. Honey Traceability

#### 3.1.1. Principal Component Analysis of Pure Honeys

**Figure 2:**
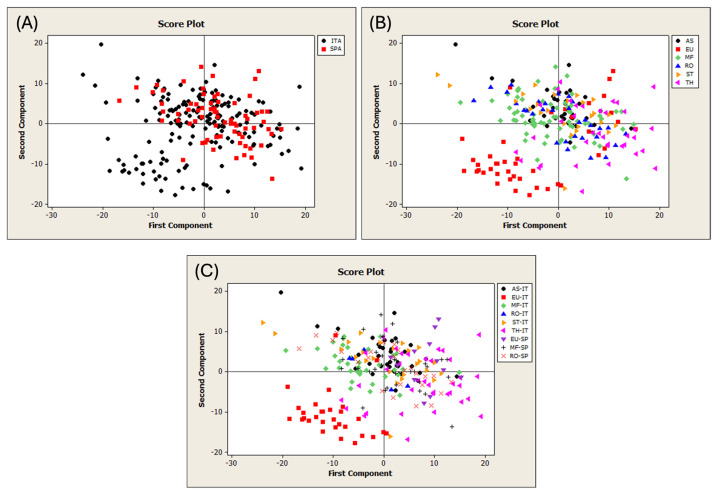
Score plots of the PCA run on the different datasets of pure honeys. (**A**) Discrimination of geographical origin; (**B**) of botanical origin; and (**C**) of both geographical and botanical origin. ITA = Sardinia; SPA = Spain; AS = asphodel; EU = eucalyptus; MF = multifloral; RO = rosemary; ST = strawberry tree; and TH = thistle.

The first PCA was performed on the FT-NIR spectra of 244 pure honey samples from Sardinia and Spain (Figure S2). The T^2^ vs. Q diagnostic plot revealed the presence of five outlier samples, which were subsequently removed from the dataset. The remaining samples were categorized based on their geographical and botanical origins. Table S4 shows the dataset division according to the type of traceability study. Figure 2 shows the PCA score plots using different spectral processing.

The samples were color-coded according to the honey categories. Figure 2A displays the geographical origin of the honeys. All the samples overlapped, except for a Sardinian group that had negative scores on PC1 and PC2. Figure 2B shows the botanical origin of the samples, which mostly overlap, except for a portion belonging to the eucalyptus category. Figure 2C highlights that this group is exclusively composed of Sardinian eucalyptus honey, which is the only category separated from the other groups. The use of different preprocessing methods did not improve the object separation. This finding is consistent with those of previous studies that examined unifloral or multifloral honey from Hungary and Argentina [37,71]. In these cases, the PCA showed no clear separation according to botanical [71] or geographical origin [37,71].

#### 3.1.2. Geographical and Botanical Classification

The dataset used for the geographical classification of honey included 168 samples from Sardinia and 71 samples from Spain for a total of 239 samples (Table S4). Variable selection was performed using the GA, whereas LDA was used to discriminate between the honeys. All the results obtained with different spectral pretreatments are reported in Table S5. The GA-selected spectral regions for each model were 926.7—957.7 nm, 1199.2—1217.8 nm, 1236.4—1248.8 nm, and 1632.8—1645.2 nm. All the mathematical transformations, except for the SNV spectral pretreatment, resulted in accurate models both in calibration and validation. The model based on the raw spectra was the most stable; it used 30 variables distributed across six spectral regions and it accurately assigned 96% of the training set samples and classified 95% of the test set samples. Table 1 displays the confusion matrix obtained using LDA for geographical classification.

The dataset was also used for botanical classification. The samples were categorized into six botanical origins (asphodel, eucalyptus, multifloral, rosemary, strawberry tree, and thistle), as shown in Table S4. The GA selected between 41 and 56 wavelengths, depending on the spectral pretreatment (Table S6). The selected spectral regions for all the models were 926.7–945.3 nm, 1001.0–1007.2 nm, 1056.8–1100.1 nm, 1124.9–1131.1 nm, 1186.8 nm, 1347.9–1354.1 nm, 1391.3–1416.0 nm, 1478.0–1509.0 nm, 1539.9–1546.1 nm, and 1601.9–1657.6 nm. The best performance was achieved using raw spectra and SNV-based models when considering the validation accuracy. The model based on raw spectra used 49 wavelengths distributed in 12 regions and correctly assigned 85% of the training samples, with an average prediction accuracy of 91%. Table 2 shows the corresponding confusion matrix obtained using LDA for botanical classification.

The dataset was then divided into nine categories based on both geographical and botanical origin (Table S4). Table S7 presents the results of the models built by selecting the spectral variables after different mathematical pretreatments. The number of wavelengths selected by the GA ranged from 36 (second-derivative MSC spectra) to 54 (first-derivative SNV spectra). The selected spectral regions were 920.5–926.7 nm, 1056.8–1063.0 nm, 1205.4–1217.8 nm, 1490.4–1509.0 nm, 1546.1–1552.3 nm, and 1657.6 nm. The raw and SNV-treated spectra led to more balanced models in terms of training and testing accuracy. The first model accurately classified 83% and 85% of the training and test set samples, respectively. Similarly, the second model correctly classified 83% of the training set and 83% of the test set honey. Table 3 presents the confusion matrix of the LDA model for the geographical–botanical classification.

The geographical model (Table 1) was more accurate than the botanical one (Table 2), whereas the accuracy tended to decrease when the origins were combined (Table 3). This finding agrees with the previous data obtained from this dataset using elemental fingerprinting [18]. As previously reported, these conditions increase the difficulties in honey traceability studies. However, comparing common botanical varieties from different geographical origins ensures that discrimination based on this factor is less significantly influenced by botanical origin. Regarding data processing, the raw spectra enabled the development of highly accurate models, thereby reducing the importance of the pretreatment method selected. However, it is essential to remark that the spectral regions identified by the genetic algorithms may vary depending on the botanical and geographical origin under consideration, as well as the necessity to use other pretreatment methods.

The results obtained can be compared with other methodologies adopted for the botanical discrimination of these varieties. This research group has spent years investigating unifloral honeys from Sardinia, employing various methodologies for botanical discrimination. The combination of four simple parameters, namely, pH, acidity, conductivity, and DPPH, enabled the discrimination of honeys with a prediction accuracy of 100% [72]. In contrast, the application of an elemental analysis [73], ATR-FTIR with random forest or genetic algorithms [33,39], discriminated honeys with an accuracy of 87%. In this study, portable NIR enabled botanical discrimination with 91% predictive accuracy, demonstrating comparable performance to previous approaches while offering the significant advantages associated with the speed and cost-effectiveness of the technique.

Regarding the geographical discrimination, although the accuracy obtained is 95%, the results should be discussed considering also the botanical origin. Looking at the results obtained combining both origins (Table 3), multifloral, eucalyptus, and rosemary honeys from Spain were rarely classified as Italian samples. The accuracy of this approach is comparable to that of other techniques, such as elemental or isotopic analysis [18,21,74], which are among the most widely used for geographic discrimination because the elements or isotopic ratios are closely related to the environment.

A review of the literature reveals that NIR spectroscopy has rarely been employed to simultaneously trace botanical and geographical origins. Furthermore, the data available are largely limited to results obtained with benchtop devices. Guelpa et al. [49] employed a portable NIR for the differentiation of imported and exported honey, yet they did not consider the impact of botanical varieties on the results. While the discrimination is accurate, the research design does not ensure that the discrimination observed is dependent on botanical or geographical origins. Bodor et al. [71] discriminated between different unifloral honeys produced in various regions of Hungary. The models were generally less accurate, and the botanical classification model was more accurate than the geographical classification model. Truong et al. [75] used visible–near-infrared spectroscopy to authenticate mono-floral, multifloral mānuka, and other (non-mānuka) honeys from eight geographic regions in New Zealand. The accuracy of the models was consistent with the results obtained in this study. However, some spectral regions selected in the 1000–1400 nm range do not match, which may be due to the different origins of the honeys investigated. The results obtained in this study are also consistent with those reported by Damiani et al. [37] and Ballabio et al. [76], who differentiated honey from various regions of Argentina and Italy using a data fusion approach. However, in this case, in addition to the NIR spectra, their models were calculated using other data obtained by FT-MIR and FT-Raman.

In conclusion, the results of this study support that portable NIR spectroscopy is a feasible screening method for tracing both the botanical and geographical origin of honey. Nevertheless, geographical classification is more accurate when considering different botanical origins that are not common to the geographical regions under consideration. Therefore, as initially assumed, traceability studies need to consider both aspects to be generalizable. The findings also confirm that unifloral honeys (such as asphodel, strawberry tree, and thistle honey) can be traced with higher accuracy than multifloral honeys. This may be attributed to the lower intraclass variability that is characteristic of unifloral honeys. As a result, on the other hand, the proposed approach and the obtained models suggest that NIR can discriminate between multifloral and unifloral honeys, which is a significant outcome given the disparate economic values.

### 3.2. Detection of Adulterants

#### 3.2.1. Principal Component Analysis of Adulterated Honeys

**Figure 3:**
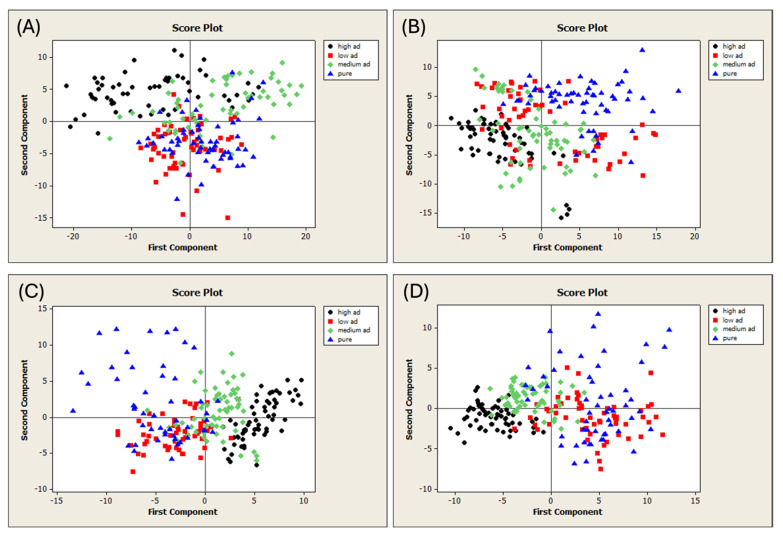
Score plots of the PCA run on the different datasets of adulterated honeys. (**A**) Dataset MFAD1, 1st-derivative MSC spectra; (**B**) Dataset MFAD2, 1st-derivative spectra; (**C**) Dataset UFAD1, 2nd-derivative SNV spectra; and (**D**) Dataset UFAD2, 2nd-derivative spectra. MF = multifloral honey; UF = unifloral honey; AD1 = first adulterant syrup; and AD2 = second adulterant syrup.

The unifloral (UF) and multifloral (MF) pure honey samples were adulterated with two different adulterant syrups (AD1 and AD2). The samples were subjected to FT-NIR spectral acquisition, resulting in four different datasets of 240 samples each (Figure 1A). PCA was performed on each dataset to detect outliers. The T^2^ vs. Q diagnostic plots obtained are shown in Figure S3. Figure 3 shows the PCA score plots, and the samples are colored according to their level of adulteration. All the spectral pretreatments were evaluated, and the plots with the best visual separation are presented in Figure 3.

The various categories were not separated despite a consistent trend in all the plots. Indeed, the pure samples were completely separated from their adulterated counterparts. This distinction was achieved by applying the following spectral pretreatments: MSC and the first derivative on the MFAD1 dataset (Figure 3A), the first derivative on the MFAD2 dataset (Figure 3B), the SNV and second derivative to the UFAD1 dataset (Figure 3C), and the second derivative to the UFAD2 dataset (Figure 3D). The PCA identified patterns in all the cases under investigation. These results are consistent with previous studies that have examined unifloral honey from China adulterated with rice syrup and corn syrup [77], acacia honey from Croatia adulterated with glucose syrup [78,79], and honey from Hungary and Spain adulterated with HFCS [38,80].

#### 3.2.2. Classification of Pure and Adulterated Honeys

LDA was used to distinguish between pure honey samples (multifloral and unifloral) and adulterated samples (spiked with AD1 and AD2 syrups). As described in Section 2.4, the models were developed separately for each dataset using various pretreatment methods and the GA for variable selection. The outliers were removed using the T^2^ vs. Q diagnostic plots (Figure S3). All the results are reported in the Supplementary Material (Tables S8–S11). Table 4 presents the performance of the best model for each dataset. The most accurate models for classifying pure and adulterated multifloral honeys were those obtained using the second-derivative MSC (MFAD1) and first-derivative MSC (MFAD2) as spectra processing. The average cross-validation accuracy was 97% when the honeys were adulterated with AD1 and 92% when adulterated with AD2. The prediction accuracies were 100% and 99%, respectively. Considering the unifloral honeys, the best results were obtained using MSC (UFAD1) and the first-derivative SNV (UFAD2). In this case, the accuracy did not vary significantly according to the type of adulterating syrup. The cross-validation accuracy was 96%, while the prediction accuracy was 98% and 100%, respectively. Notably, the LDA-MFAD1 model was more accurate than LDA-MFAD2, whereas LDA-UFAD2 was more accurate than LDA-UFAD1. Overall, the performance of the models depended on the botanical origin and adulterant used. Multifloral honey may have a higher variability in the fructose/glucose ratio, which can make identification more difficult when honeys are adulterated with AD2 syrups. In contrast, unifloral honey has a less variable composition, making adulteration relatively easier to detect.

#### 3.2.3. Quantification of Adulterants by PLS

The percentage of adulterants in the multifloral and unifloral honey was estimated using PLS. As described in Section 2.4, the models were built considering the two adulterant syrups and multifloral and unifloral honeys separately, resulting in four datasets. The outliers were removed using T^2^ vs. Q diagnostic plots (Figure S3). The performance was evaluated based on the different pretreatment methods, using both GA-selected variables and full spectra. All the results are reported in the Supplementary Material (Tables S12–S15). Generally, for each dataset, the best preprocessing method was GA-MSC.

**Figure 4:**
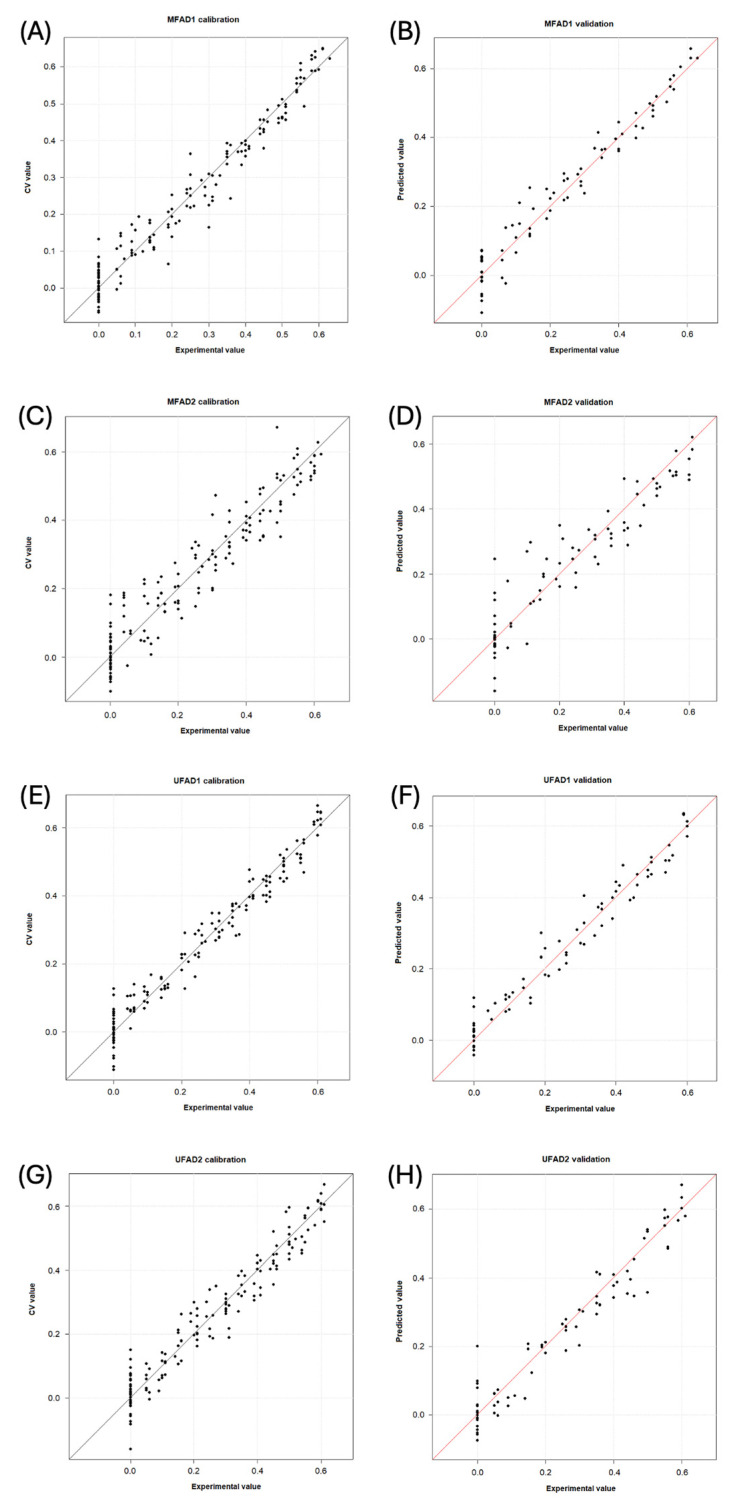
Experimental versus calculated value plots obtained by the models for detecting adulterants in multifloral and unifloral honeys (percentages of adulteration are shown as decimal). (**A**) MFAD1, calibration step; (**B**) MFAD1, validation step; (**C**) MFAD2, calibration step; (**D**) MFAD2, validation step; (**E**) UFAD1, calibration step; (**F**) UFAD1, validation step; (**G**) UFAD2, calibration step; and (**H**) UFAD2, validation step. MF = multifloral honey; UF = unifloral honey; AD1 = first adulterant syrup; and AD2 = second adulterant syrup.

Table 5 presents the performance of each PLS model. Figure 4 displays for each dataset the ‘predicted vs. measured’ graphics in calibration and prediction, respectively.

The MFAD1 model is characterized by an explained variance of 95.8% using nine latent variables and 13 wavelengths. The root mean square error of cross-validation (RMSECV) was 4% for a median adulterant/honey (A/H) value of 32% (relative error of 13%). The root mean square error of prediction (RMSEP) was 4% for a median A/H value of 34% (relative error of 13%). The samples with adulteration levels above 15% A/H were accurately predicted, whereas those below 10% were frequently detected as pure honey (Figure 4A,B). The MFAD2 model used 35 wavelengths distributed across five spectral regions, which described 90.4% of the total variance using 10 latent variables. The RMSECV was 6% for a median value of 30% A/H (relative error of 20%), and the RMSEP was 7% for a median value of 31% A/H (relative error of 23%). The samples containing 20% A/H or more were correctly identified as adulterated, whereas the samples with lower concentrations could be mistaken for pure honey (Figure 4C,D).

The UFAD1 model demonstrated consistent performance, with an explained variance of 96.1% using 10 latent variables and 19 wavelengths. The RMSECV was 4% for a median value of 33% A/H (relative error of 12%). The RMSEP was 4% for a median value of 33% A/H (relative error of 12%). The model allowed for the accurate prediction of the adulteration levels at concentrations of 15% A/H or higher (Figure 4E,F). Finally, for the UFAD2 dataset, the most promising model was the GA first-derivative MSC spectrum-based model, which used 28 wavelengths distributed across seven spectral regions. It was characterized by eight latent variables that described 93.9% of the total variance. The RMSECV was 5% for a median value of 35% A/H (relative error of 14%). The RMSEP was 5% for a median value of 31% A/H (relative error of 16%). Except for one pure sample, which was predicted to contain 20 wt% A/H, every sample was accurately estimated. The pure samples were only confused with honeys adulterated at A/H < 10% (Figure 4G,H).

Previous studies on acacia honey from Croatia reported a similar accuracy of PLS models in predicting honey adulterated with fructose and glucose syrups [78,79]. The authors found that Artificial Neural Networks performed better than PLS, but implementing this algorithm in management software may be challenging for routine analysis. Li et al. [81] developed qualitative and quantitative methods to detect HFCS and maltose syrup in 12 unifloral honeys from China. The results were in excellent agreement with those obtained for multifloral honeys from Spain that were adulterated with rice syrup, inverted sugar, brown cane sugar, fructose syrup, and HFCS [38,82].

The results obtained using PLS regression support what was observed using LDA. The PLS-MFAD1 model accurately predicted adulterated samples with AD > 10% A/H, whereas the PLS-MFAD2 model correctly predicted samples with AD > 20% A/H. In contrast, PLS-UFAD1 and PLS-UFAD2 had limits of 15% and 10% A/H, respectively. Therefore, it is easier to quantify adulterants in unifloral than multifloral honeys. The findings of this study also confirm that portable NIR can be employed for adulterant quantification, with levels of prediction similar to those obtained with benchtop devices.

## 4. Conclusions

This study proposes and validates the use of portable NIR spectroscopy for honey screening. It allows for the prediction of honey origin and the detection of adulterants through fast and on-site analysis. Qualitative and quantitative models were developed for both unifloral and multifloral honey samples. Two of the most common adulterants with different compositions of glucose, fructose, and maltose were evaluated. The data were processed using several pretreatment methods, including full-spectrum analysis and genetic algorithms for variable selection. The preprocessing methods had a minimal impact on the accuracy of the models, although multiplicative scatter correction was generally the most effective. The models were developed using common and user-friendly chemometric tools that can be easily implemented in quality control software. Based on the results obtained, the botanical origin influences the ability of each model to predict adulterants. Similarly, the botanical origin also affects geographical classification.

This study shows that portable NIR can be used to detect adulterants and predict botanical and geographical origin. Notably, models need to be developed considering both factors; otherwise, the accuracy of the origin prediction may be biased. As requested by the European Community, this approach aims to identify counterfeit honey quickly, accurately, and on site. Because the analytical technique and data processing are simple, the approach could be used by regulators as well as distributors or companies. A hypothetical data processing workflow could include a first step to evaluate botanical and geographical origins, followed by a second step to identify adulterants. In the future, the datasets will be expanded to include new botanical varieties, countries of origin, and adulterants. In addition, other models will be developed to predict chemical–physical parameters and the presence of contaminants for complete quality control.

## Figures and Tables

**Table 1:** Confusion matrices of the LDA model for the geographical origin classification.

Cross-Validation				Prediction			
	ITA	SPA	Accuracy		ITA	SPA	Accuracy
ITA	108	8	93%	ITA	49	3	94%
SPA	1	46	98%	SPA	1	23	96%
Average accuracy			96%	Average accuracy			95%

ITA = Sardinia (Italy); SPA = Spain. Model: GA-raw spectra.

**Table 2:** Confusion matrices of the LDA model for the botanical origin classification.

Cross-Validation								Prediction							
	AS	EU	MF	RO	ST	TH	Accuracy		AS	EU	MF	RO	ST	TH	Accuracy
AS	22	0	1	0	0	1	92%	AS	10	0	2	0	0	0	83%
EU	1	20	2	3	2	0	71%	EU	0	13	1	0	0	0	93%
MF	4	2	35	4	0	0	78%	MF	0	0	23	0	0	0	100%
RO	1	1	3	15	0	0	75%	RO	0	0	2	9	0	0	82%
ST	0	0	0	0	18	0	100%	ST	0	1	0	0	6	0	86%
TH	0	1	0	0	0	24	96%	TH	0	0	0	0	0	12	100%
Average accuracy							85%	Average accuracy							91%

AS = asphodel; EU = eucalyptus; MF = multifloral; RO = rosemary; ST = strawberry tree; and TH = thistle. Model: GA-raw spectra.

**Table 3:** Confusion matrices of the LDA model for the botanical-geographical origin classification.

**Cross-Validation**										
	AS-IT	EU-IT	MF-IT	RO-IT	ST-IT	TH-IT	EU-SP	MF-SP	RO-SP	Accuracy
AS-IT	20	0	2	0	0	1	0	1	0	83%
EU-IT	0	20	0	0	0	0	0	0	0	100%
MF-IT	2	0	19	0	0	0	0	1	1	83%
RO-IT	0	0	1	3	0	0	0	0	0	75%
ST-IT	0	0	0	0	13	2	0	1	0	81%
TH-IT	0	1	0	0	3	21	0	0	0	84%
EU-SP	1	0	0	0	0	1	6	0	1	67%
MF-SP	2	0	1	0	0	0	0	17	2	77%
RO-SP	0	0	0	0	0	0	1	1	15	88%
Average accuracy										83%
**Prediction**										
	AS-IT	EU-IT	MF-IT	RO-IT	ST-IT	TH-IT	EU-SP	MF-SP	RO-SP	Accuracy
AS-IT	9	0	0	0	1	1	0	1	0	75%
EU-IT	2	8	0	0	0	0	0	0	0	80%
MF-IT	0	0	12	0	0	0	0	0	0	100%
RO-IT	0	0	0	1	0	0	0	0	0	100%
ST-IT	0	0	0	0	7	1	0	1	0	78%
TH-IT	0	0	0	0	1	11	0	0	0	92%
EU-SP	0	0	0	0	0	0	3	1	0	75%
MF-SP	0	0	2	0	0	0	0	8	1	73%
RO-SP	0	0	0	0	0	0	0	1	8	89%
Average accuracy										85%

IT = Sardinia (Italy); SP = Spain; AS = asphodel; EU = eucalyptus; MF = multifloral; RO = rosemary; ST = strawberry tree; and TH = thistle. Model: GA-raw spectra.

**Table 4:** Confusion matrices of LDA models for classifying adulterated honeys.

**MFAD1, 2nd-Derivative MSC Model**						**MFAD2, 1st-Derivative MSC Model**					
**Cross-Validation**						**Cross-Validation**					
Adulteration	Pure	Low	Medium	High	Accuracy	Adulteration	Pure	Low	Medium	High	Accuracy
Pure	36	3	0	0	92%	Pure	36	2	1	0	92%
Low	0	38	1	0	97%	Low	0	35	3	0	92%
Medium	0	0	38	0	100%	Medium	0	2	35	3	88%
High	0	0	0	40	100%	High	0	0	1	39	98%
Average accuracy					97%	Average accuracy					92%
**Prediction**						**Prediction**					
	Pure	Low	Medium	High	Accuracy		Pure	Low	Medium	High	Accuracy
Pure	20	0	0	0	100%	Pure	20	0	0	0	100%
Low	0	20	0	0	100%	Low	0	19	0	0	100%
Medium	0	0	20	0	100%	Medium	0	0	19	1	95%
High	0	0	0	18	100%	High	0	0	0	20	100%
Average accuracy					100%	Average accuracy					99%
**UFAD1, MSC Model**						**UFAD2, 1st-Derivative SNV Model**					
**Cross-Validation**						**Cross-Validation**					
Adulteration	Pure	Low	Medium	High	Accuracy	Adulteration	Pure	Low	Medium	High	Accuracy
Pure	33	0	0	0	100%	Pure	35	1	0	0	97%
Low	1	35	2	0	92%	Low	0	38	1	0	97%
Medium	0	0	37	0	100%	Medium	0	2	36	1	92%
High	0	0	1	40	98%	High	0	0	0	40	100%
Average accuracy					96%	Average accuracy					96%
**Prediction**						**Prediction**					
	Pure	Low	Medium	High	Accuracy		Pure	Low	Medium	High	Accuracy
Pure	16	0	0	0	100%	Pure	18	0	0	0	100%
Low	0	18	1	0	95%	Low	0	19	0	0	100%
Medium	0	1	20	0	95%	Medium	0	0	20	0	100%
High	0	0	0	19	100%	High	0	0	0	20	100%
Average accuracy					98%	Average accuracy					100%

MF = multifloral honey; UF = unifloral honey; AD1 = first adulterant syrup; and AD2 = second adulterant syrup.

**Table 5:** Prediction parameters for detecting adulterants in honey using PLS.

Dataset	Spectral Pretreatment	Variable Selection	n. of Variables	LV	Explained Variance (%)	Cross-Validation			Prediction			
						Median	RMSECV	RMSECV%	Median	RMSEP	RMSEP%	Bias
MFAD1	MSC	GA	13	9	95.8	0.32	0.04	13	0.34	0.04	13	0.006
MFAD2	MSC	GA	35	10	90.4	0.30	0.06	20	0.31	0.07	23	−0.004
UFAD1	MSC	GA	19	10	96.1	0.33	0.04	12	0.33	0.04	12	0.005
UFAD2	MSC + 1st derivative	GA	28	8	93.9	0.35	0.05	14	0.31	0.05	16	−0.008

MF = multifloral honey; UF = unifloral honey; AD1 = first adulterant syrup; AD2 = second adulterant syrup; MSC = multiplicative scatter correction; GA = genetic algorithm; LV = latent variables; RMSECV = root mean square error in cross-validation; and RMSEP = root mean square error in prediction.

## Data Availability

The original contributions presented in the study are included in the article/Supplementary Material, further inquiries can be directed to the corresponding author.

## Supplementary Materials

The following supporting information can be downloaded at: https://www.mdpi.com/article/10.3390/foods13193062/s1, Section S1: Spectral analysis; Figure S1: (a) Overlapping of the FT-NIR spectra of the honey samples. (b) Averaged spectra of each botanical honey category; Figure S2: T^2^ vs. Q diagnostic plots of the PCA calculated on the dataset of all the pure honey samples; Figure S3: T^2^ vs. Q diagnostic plots of the PCA calculated on the four adulterant datasets; (a) MFAD1; (b) MFAD2; (c) UFAD1; and (d) UFAD2; Table S1: Melissopalynological analysis of multifloral and unifloral honey from Spain and Sardinia, Italy; Table S2: Experimental plan for preparation of multifloral honeys adulterated with syrups and dataset generation; Table S3: Experimental plan for preparation of unifloral honeys adulterated with syrups and dataset generation; Table S4: Division of the honey dataset into the different categories according to traceability; Table S5: Discrimination of honeys based on geographical origin using LDA; Table S6: Discrimination of honeys based on botanical origin using LDA; Table S7: Discrimination of honeys based on geographical–botanical origin using LDA; Table S8: Classification parameters for detecting AD1 adulteration in multifloral honeys using LDA with varying spectral pretreatments; Table S9: Classification parameters for detecting AD2 adulteration in multifloral honeys using LDA with varying spectral pretreatments; Table S10: Classification parameters for detecting AD1 adulteration in unifloral honeys using LDA with varying spectral pretreatments; Table S11: Classification parameters for detecting AD2 adulteration in unifloral honeys using LDA with varying spectral pretreatments; Table S12: Prediction parameters for detecting AD1 adulteration in honeys using PLS with varying spectral pretreatments; Table S13: Prediction parameters for detecting AD2 adulteration in multifloral honeys using PLS with varying spectral pretreatments; Table S14: Prediction parameters for detecting AD1 adulteration in unifloral honeys using PLS with varying spectral pretreatments; Table S15: Prediction parameters for detecting AD2 adulteration in unifloral honeys using PLS with varying spectral pretreatments [65,66,67,68,69].
