# Supplementary material for: Portable NIR Spectroscopy to Simultaneously Trace Honey Botanical and Geographical Origins and Detect Syrup Adulteration
Source: Foods. 2024 Sep 26;13(19):3062. doi: 10.3390/foods13193062 (PMC11476024; doi:10.3390/foods13193062)
Supplement: Supplementary file 1 [file foods-13-03062-s001.zip › foods-3218057-supplementary.pdf]

Portable NIR spectroscopy to simultaneously trace honey botanical and geographical origins and detect syrup adulteration

Marco Caredda, Marco Ciulu, Francesca Tilocca, Ilaria Langasco, Oscar Núñez, Sònia Sentellas, Javier Saurina Maria I. Pilo, Nadia Spano, Gavino Sanna*, Andrea Mara*

amara@uniss.it; sanna@uniss.it

Abstract: Fraudulent practices concerning honey are growing fast and involve misrepresentation of origin and adulteration. Simple and feasible methods for honey authentication are needed to ascertain honey compliance and quality. Working on a robust dataset and simultaneously investigating honey traceability and adulterant detection, this study proposed a portable FTNIR fingerprinting approach combined with chemometrics. Multifloral and unifloral honey samples (n=244) from Spain and Sardinia (Italy) were discriminated by botanical and geographical origin. Qualitative and quantitative methods were developed using Linear Discriminant Analysis (LDA) and Partial Least Square (PLS) regression to detect adulterated honey with two syrups, consisting of glucose, fructose, and maltose. Botanical and geographical origins were predicted with accuracies of 90% and 95%, respectively. LDA models discriminated pure and adulterated honey samples with an accuracy of over 92%, whereas PLS allows the accurate quantification of over 10% of adulterants in unifloral and 20% in multifloral honey.

Keywords: Honey; Near-infrared spectroscopy; Adulteration; Geographical origin; Botanical origin

SUPPLEMENTARY MATERIAL

S1. Spectral Analysis

The NIR spectral region, spanning from 800 nm to 2500 nm, is characterized by absorptions resulting from overtones and combinations of stretching modes of vibrations of molecules that typically contain C-H, N-H, or O-H bonds. Spectroscopists have traditionally avoided this region due to its challenging interpretation compared to the mid-infrared region, as NIR bands often overlap and spectra exhibit broad peaks (Irudayaraj & Reh, 2008; Rodriguez-Otero et al., 1997). In general, combination bands of two or more simultaneous vibrations are typically found in the regions 1300 to 1420 nm (C-H stretching) and 2000 to 2500 nm (C-H, N-H and O-H stretchings). First overtones of the same chemical bonds can be observed in the regions from 1400 nm to 1500 nm (N-H and O-H stretchings) and from 1600 nm to 1800 nm (C-H stretching), while the second overtones lie in the regions from 950 nm to 1100 nm (N-H and O-H stretchings) and from 1100 nm to 1225 nm (C-H stretching). Finally, the third overtones occur in the regions from 775 nm to 850 nm (N-H stretching) and from 850 nm to 950 nm (C-H stretching) (Bittante et al., 2022; Schwanninger et al., 2011; Stuart, 2004). The FT-NIR spectral region used in this study for honey analysis is between 908 nm and 1676 nm. Only the bands from the second overtone are completely included in this region, while those from the first and the third overtones, as well as the combination bands, are here only partially reported. The informative spectral regions selected by GA for the different studies of this work were mainly in the regions from 914.3 nm to 957.7 nm (third overtone of C-H stretching), from 1001.0 nm to 1025.8 nm and from 1044.4 nm to 1137.3 nm (second overtone of C-H, N-H and O-H stretchings), from 1193.0 nm to 1255.0 nm (second overtone of C-H stretching),from 1310.7 nm to 1360.3 nm (combination of C–H stretching), and from 1601.9 nm to 1657.6 nm (part of first overtone of C–H stretching).

1. Rodriguez-Otero, J.L.; Hermida, M.; Centeno, J. Analysis of Dairy Products by Near-Infrared Spectroscopy: A Review. J. Agric. Food Chem. 1997, 45, 2815–2819, doi:10.1021/jf960744p.

2. Nondestructive Testing of Food Quality; Irudayaraj, J., Reh, C., Eds.; IFT Press series; 1st ed.; Blackwell Pub. ; IFT Press: Ames, Iowa, 2008; ISBN 978-0-8138-2885-5.

3. Bittante, G.; Patel, N.; Cecchinato, A.; Berzaghi, P. Invited Review: A Comprehensive Review of Visible and near-Infrared Spectroscopy for Predicting the Chemical Composition of Cheese. Journal of Dairy Science 2022, 105, 1817–1836, doi:10.3168/jds.2021-20640.

4. Stuart, B.H. Infrared Spectroscopy: Fundamentals and Applications; Analytical Techniques in the Sciences; 1st ed.; Wiley, 2004; ISBN 978-0-470-85427-3.

5. Schwanninger, M.; Rodrigues, J.C.; Fackler, K. A Review of Band Assignments in near Infrared Spectra of Wood and Wood Components. Journal of Near Infrared Spectroscopy 2011, 19, 287–308, doi:10.1255/jnirs.955.

Figure S1. a) Overlapping of the FT-NIR spectra of the honey samples. b) Averaged spectra of each botanical honey category.

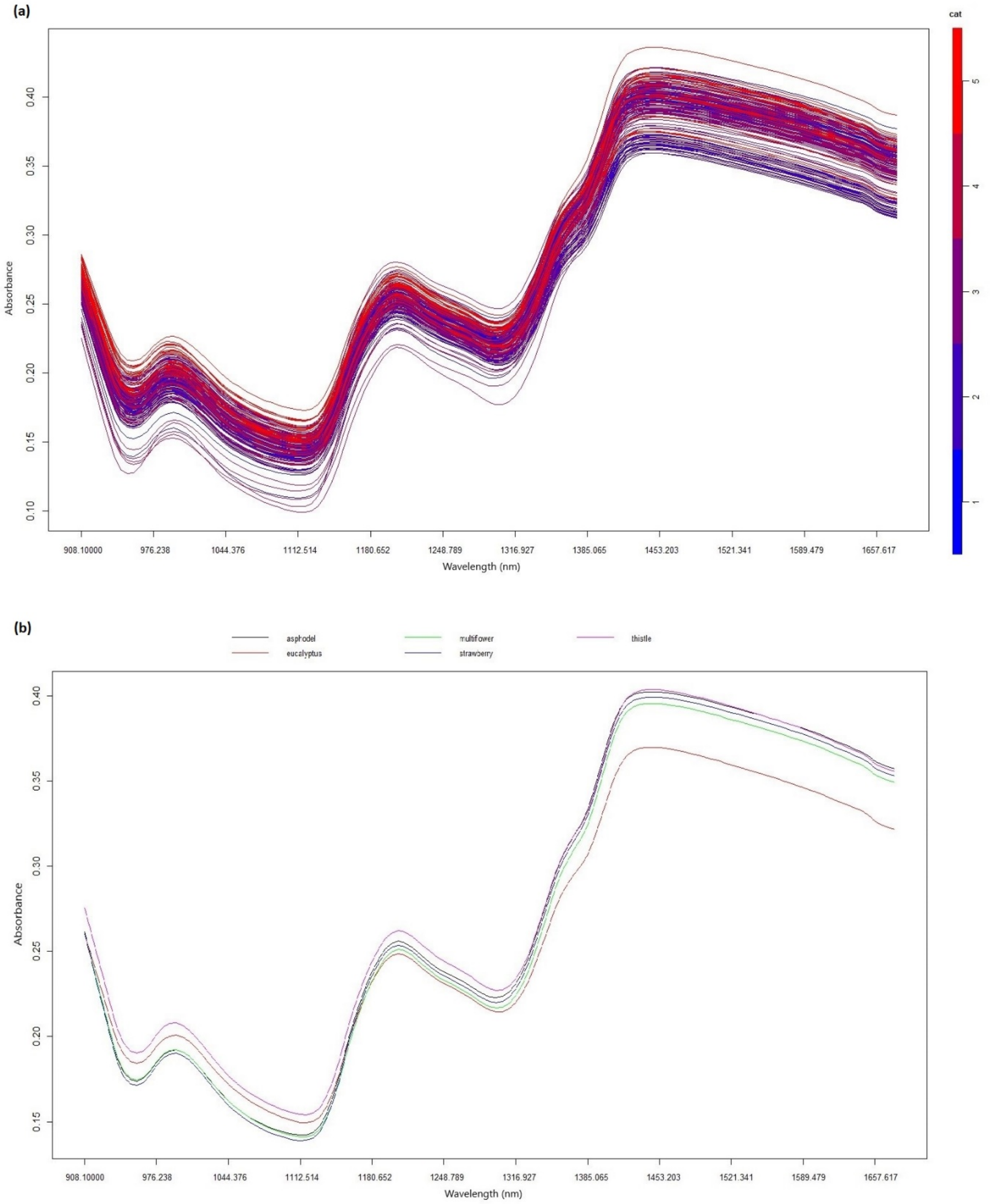

Colors refer to the botanical origin: blue = asphodel; violet = eucalyptus; purple = multifloral; brown = strawberry; red = thistle.

Figure S2. T^2 vs Q diagnostic plots of the PCA calculated on the dataset of all the pure honey samples.

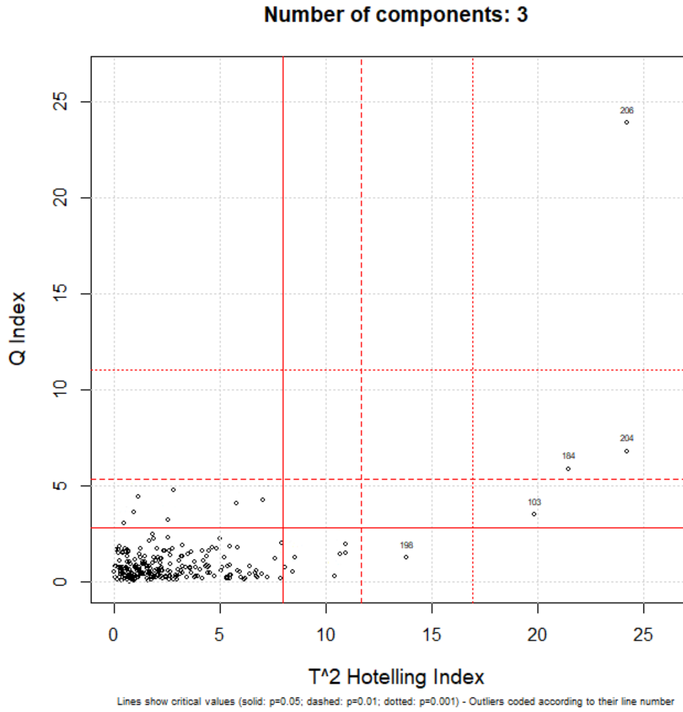

Figure S3. T^2 vs Q diagnostic plots of the PCA calculated on the four adulterant datasets; a) MFAD1; b) MFAD2; c) UFAD1; d) UFAD2.

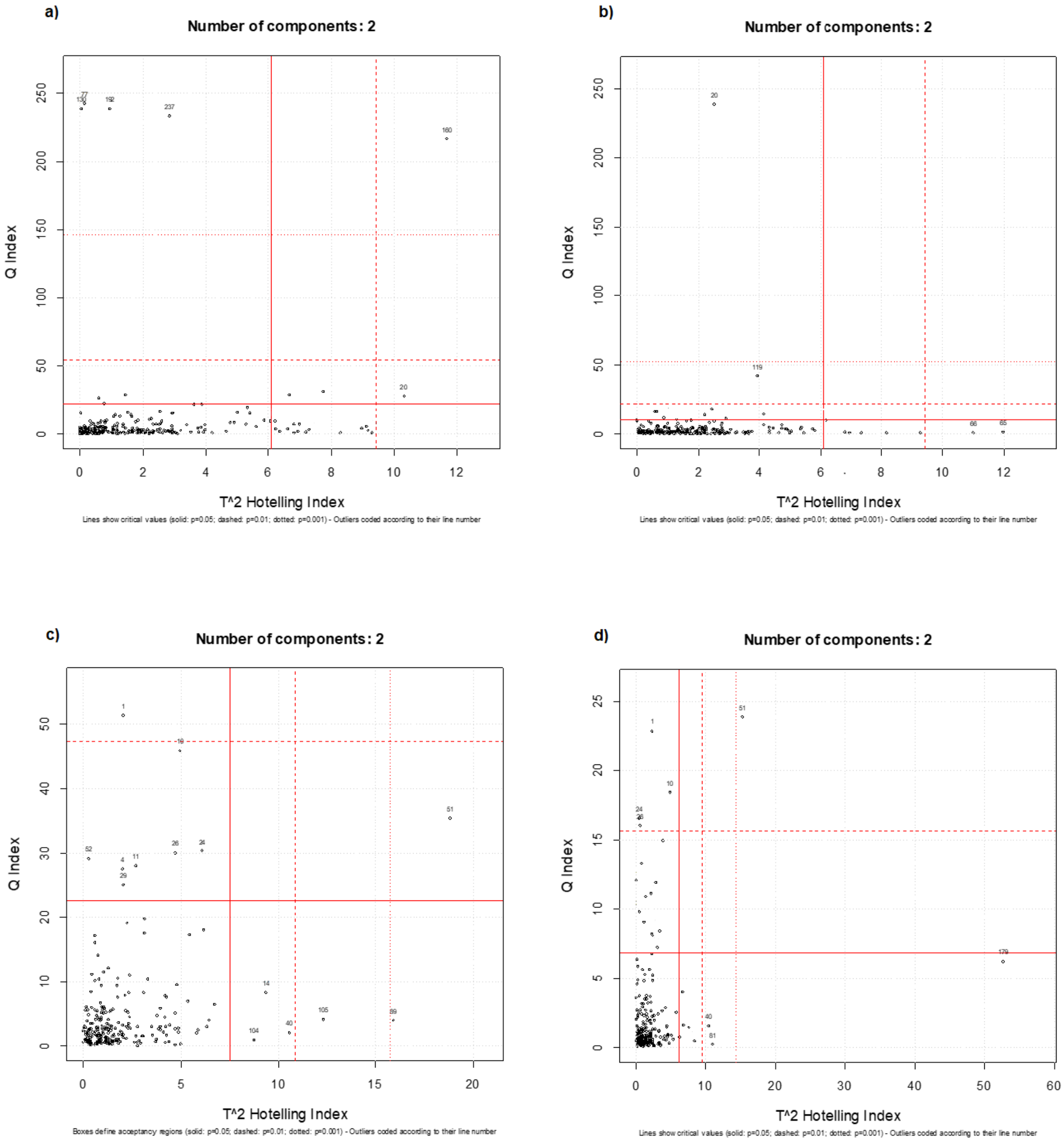

MF = multifloral honey; UF = unifloral honey; AD1 = first adulterant syrups; AD2 = second adulterant syrups

Table S1. Melissopalynological analysis of multifloral and unifloral honey from Spain and Sardinia, Italy.

Country of origin	Botanical species	Number of samples	Pollen speciess	Threshold for the classification (pollen %)
Spain	Multifloral	34	*Vary according to seasonality and provenance, some common pollens are: Salvia Rosmarinus, Echium plantagineum L. Cistus spp, Erica spp, Castanea sativa Miller, Lavandula stoechas L., and Eucalyptus sp.*	No prevalent pollen species
	Rosemary	26	*Salvia rosmarinus*	> 10 %
	Eucalyptus	13	*Eucalyptus camaldulensis, E. globulus, and E. viminalis*	> 90 %
Italy	Multifloral	35	*Vary according to seasonality and provenance, some common pollens are Asfodelus spp., Echium spp., Trifolium spp., Rosmarinus Galactites tormentosa, and Cistus spp*	No prevalent pollen species
	Rosemary	7	*Salvia rosmarinus*	> 10 %
	Eucalyptus	28	*Eucalyptus camaldulensis, E. globulus, and E. viminalis*	> 90 %
	Asphodel		*Asphodelus microcarpus and other Asphodelus spp.*	> 7 %
	Thistle		*Galactites tomentosa, Silybum marianum, Cirsium, Centaurea, Carlina, Carduus and Cynara*	> 5 %
	Strawberry-tree		*Arbutus unedo*	> 7 %

Table S2. Experimental plan for preparation of multifloral honeys adulterated with syrups and dataset generation.

Pure Honey	Low Adulteration				Medium Adulteration				High adulteration			
0%	5%	10%	15%	20%	25%	30%	35%	40%	45%	50%	55%	60%
IT-MF01_AD1_0	IT-MF01_AD1_5				IT-MF01_AD1_25				IT-MF01_AD1_45			
IT-MF02_AD1_0		IT-MF02_AD1_10				IT-MF02_AD1_30				IT-MF02_AD1_50		
IT-MF03_AD1_0			IT-MF03_AD1_15				IT-MF03_AD1_35				IT-MF03_AD1_55	
IT-MF04_AD1_0				IT-MF04_AD1_20				IT-MF04_AD1_40				IT-MF04_AD1_60
IT-MF05_AD1_0	IT-MF05_AD1_5				IT-MF05_AD1_25				IT-MF05_AD1_45			
IT-MF06_AD1_0		IT-MF06_AD1_10				IT-MF06_AD1_30				IT-MF06_AD1_50		
IT-MF07_AD1_0			IT-MF07_AD1_15				IT-MF07_AD1_35				IT-MF07_AD1_55	
IT-MF08_AD1_0				IT-MF08_AD1_20				IT-MF08_AD1_40				IT-MF08_AD1_60
IT-MF09_AD1_0	IT-MF09_AD1_5				IT-MF09_AD1_25				IT-MF09_AD1_45			
IT-MF10_AD1_0		IT-MF10_AD1_10				IT-MF10_AD1_30				IT-MF10_AD1_50		
IT-MF11_AD1_0			IT-MF11_AD1_15				IT-MF11_AD1_35				IT-MF11_AD1_55	
IT-MF12_AD1_0				IT-MF12_AD1_20				IT-MF12_AD1_40				IT-MF12_AD1_60
IT-MF13_AD1_0	IT-MF13_AD1_5				IT-MF13_AD1_25				IT-MF13_AD1_45			
IT-MF14_AD1_0		IT-MF14_AD1_10				IT-MF14_AD1_30				IT-MF14_AD1_50		
IT-MF15_AD1_0			IT-MF15_AD1_15				IT-MF15_AD1_35				IT-MF15_AD1_55	
IT-MF16_AD1_0				IT-MF16_AD1_20				IT-MF16_AD1_40				IT-MF16_AD1_60
IT-MF17_AD1_0	IT-MF17_AD1_5				IT-MF17_AD1_25				IT-MF17_AD1_45			
IT-MF18_AD1_0		IT-MF18_AD1_10				IT-MF18_AD1_30				IT-MF18_AD1_50		
IT-MF19_AD1_0			IT-MF19_AD1_15				IT-MF19_AD1_35				IT-MF19_AD1_55	
IT-MF20_AD1_0				IT-MF20_AD1_20				IT-MF20_AD1_40				IT-MF20_AD1_60
IT-MF21_AD1_0	IT-MF21_AD1_5				IT-MF21_AD1_25				IT-MF21_AD1_45			
IT-MF22_AD1_0		IT-MF22_AD1_10				IT-MF22_AD1_30				IT-MF22_AD1_50		
IT-MF23_AD1_0			IT-MF23_AD1_15				IT-MF23_AD1_35				IT-MF23_AD1_55	
IT-MF24_AD1_0				IT-MF24_AD1_20				IT-MF24_AD1_40				IT-MF24_AD1_60
IT-MF25_AD1_0	IT-MF25_AD1_5				IT-MF25_AD1_25				IT-MF25_AD1_45			
IT-MF26_AD1_0		IT-MF26_AD1_10				IT-MF26_AD1_30				IT-MF26_AD1_50		
IT-MF27_AD1_0			IT-MF27_AD1_15				IT-MF27_AD1_35				IT-MF27_AD1_55	
IT-MF28_AD1_0				IT-MF28_AD1_20				IT-MF28_AD1_40				IT-MF28_AD1_60
IT-MF29_AD1_0	IT-MF29_AD1_5				IT-MF29_AD1_25				IT-MF29_AD1_45			
IT-MF30_AD1_0		IT-MF30_AD1_10				IT-MF30_AD1_30				IT-MF30_AD1_50		
SP-MF01_AD1_0			SP-MF01_AD1_15				SP-MF01_AD1_35				SP-MF01_AD1_55	
SP-MF02_AD1_0				SP-MF02_AD1_20				SP-MF02_AD1_40				SP-MF02_AD1_60
SP-MF03_AD1_0	SP-MF03_AD1_5				SP-MF03_AD1_25				SP-MF03_AD1_45			
SP-MF04_AD1_0		SP-MF04_AD1_10				SP-MF04_AD1_30				SP-MF04_AD1_50		
SP-MF05_AD1_0			SP-MF05_AD1_15				SP-MF05_AD1_35				SP-MF05_AD1_55	
SP-MF06_AD1_0				SP-MF06_AD1_20				SP-MF06_AD1_40				SP-MF06_AD1_60
SP-MF07_AD1_0	SP-MF07_AD1_5				SP-MF07_AD1_25				SP-MF07_AD1_45			
SP-MF08_AD1_0		SP-MF08_AD1_10				SP-MF08_AD1_30				SP-MF08_AD1_50		
SP-MF09_AD1_0			SP-MF09_AD1_15				SP-MF09_AD1_35				SP-MF09_AD1_55	
SP-MF10_AD1_0				SP-MF10_AD1_20				SP-MF10_AD1_40				SP-MF10_AD1_60
SP-MF11_AD1_0	SP-MF11_AD1_5				SP-MF11_AD1_25				SP-MF11_AD1_45			
SP-MF12_AD1_0		SP-MF12_AD1_10				SP-MF12_AD1_30				SP-MF12_AD1_50		
SP-MF13_AD1_0			SP-MF13_AD1_15				SP-MF13_AD1_35				SP-MF13_AD1_55	
SP-MF14_AD1_0				SP-MF14_AD1_20				SP-MF14_AD1_40				SP-MF14_AD1_60
SP-MF15_AD1_0	SP-MF15_AD1_5				SP-MF15_AD1_25				SP-MF15_AD1_45			
SP-MF16_AD1_0		SP-MF16_AD1_10				SP-MF16_AD1_30				SP-MF16_AD1_50		
SP-MF17_AD1_0			SP-MF17_AD1_15				SP-MF17_AD1_35				SP-MF17_AD1_55	
SP-MF18_AD1_0				SP-MF18_AD1_20				SP-MF18_AD1_40				SP-MF18_AD1_60
SP-MF19_AD1_0	SP-MF19_AD1_5				SP-MF19_AD1_25				SP-MF19_AD1_45			
SP-MF20_AD1_0		SP-MF20_AD1_10				SP-MF20_AD1_30				SP-MF20_AD1_50		
SP-MF21_AD1_0			SP-MF21_AD1_15				SP-MF21_AD1_35				SP-MF21_AD1_55	
SP-MF22_AD1_0				SP-MF22_AD1_20				SP-MF22_AD1_40				SP-MF22_AD1_60
SP-MF23_AD1_0	SP-MF23_AD1_5				SP-MF23_AD1_25				SP-MF23_AD1_45			
SP-MF24_AD1_0		SP-MF24_AD1_10				SP-MF24_AD1_30				SP-MF24_AD1_50		
SP-MF25_AD1_0			SP-MF25_AD1_15				SP-MF25_AD1_35				SP-MF25_AD1_55	
SP-MF26_AD1_0				SP-MF26_AD1_20				SP-MF26_AD1_40				SP-MF26_AD1_60
SP-MF27_AD1_0	SP-MF27_AD1_5				SP-MF27_AD1_25				SP-MF27_AD1_45			
SP-MF28_AD1_0		SP-MF28_AD1_10				SP-MF28_AD1_30				SP-MF28_AD1_50		
SP-MF29_AD1_0			SP-MF29_AD1_15				SP-MF29_AD1_35				SP-MF29_AD1_55	
SP-MF30_AD1_0				SP-MF30_AD1_20				SP-MF30_AD1_40				SP-MF30_AD1_60

Table reports the case of AD1 as example. IT = Sardinia (Italy); SP = Spain; MF = Multifloral; AD1 = first adulterant

Table S3. Experimental plan for preparation of unifloral honeys adulterated with syrups and dataset generation.

Pure Honey	Low Adulteration				Medium Adulteration				High adulteration			
0%	5%	10%	15%	20%	25%	30%	35%	40%	45%	50%	55%	60%
TH01_AD2_0	TH01_AD2_5				TH01_AD2_25				TH01_AD2_45			
TH02_AD2_0		TH02_AD2_10				TH02_AD2_30				TH02_AD2_50		
TH03_AD2_0			TH03_AD2_15				TH03_AD2_35				TH03_AD2_55	
TH04_AD2_0				TH04_AD2_20				TH04_AD2_40				TH04_AD2_60
TH05_AD2_0	TH05_AD2_5				TH05_AD2_25				TH05_AD2_45			
TH06_AD2_0		TH06_AD2_10				TH06_AD2_30				TH06_AD2_50		
TH07_AD2_0			TH07_AD2_15				TH07_AD2_35				TH07_AD2_55	
TH08_AD2_0				TH08_AD2_20				TH08_AD2_40				TH08_AD2_60
TH09_AD2_0	TH09_AD2_5				TH09_AD2_25				TH09_AD2_45			
TH10_AD2_0		TH10_AD2_10				TH10_AD2_30				TH10_AD2_50		
TH11_AD2_0			TH11_AD2_15				TH11_AD2_35				TH11_AD2_55	
TH12_AD2_0				TH12_AD2_20				TH12_AD2_40				TH12_AD2_60
TH13_AD2_0	TH13_AD2_5				TH13_AD2_25				TH13_AD2_45			
TH14_AD2_0		TH14_AD2_10				TH14_AD2_30				TH14_AD2_50		
TH15_AD2_0			TH15_AD2_15				TH15_AD2_35				TH15_AD2_55	
EU01_AD2_0				EU01_AD2_20				EU01_AD2_40				EU01_AD2_60
EU02_AD2_0	EU02_AD2_5				EU02_AD2_25				EU02_AD2_45			
EU03_AD2_0		EU03_AD2_10				EU03_AD2_30				EU03_AD2_50		
EU04_AD2_0			EU04_AD2_15				EU04_AD2_35				EU04_AD2_55	
EU05_AD2_0				EU05_AD2_20				EU05_AD2_40				EU05_AD2_60
EU06_AD2_0	EU06_AD2_5				EU06_AD2_25				EU06_AD2_45			
EU07_AD2_0		EU07_AD2_10				EU07_AD2_30				EU07_AD2_50		
EU08_AD2_0			EU08_AD2_15				EU08_AD2_35				EU08_AD2_55	
EU09_AD2_0				EU09_AD2_20				EU09_AD2_40				EU09_AD2_60
EU10_AD2_0	EU10_AD2_5				EU10_AD2_25				EU10_AD2_45			
EU11_AD2_0		EU11_AD2_10				EU11_AD2_30				EU11_AD2_50		
EU12_AD2_0			EU12_AD2_15				EU12_AD2_35				EU12_AD2_55	
EU13_AD2_0				EU13_AD2_20				EU13_AD2_40				EU13_AD2_60
EU14_AD2_0	EU14_AD2_5				EU14_AD2_25				EU14_AD2_45			
EU15_AD2_0		EU15_AD2_10				EU15_AD2_30				EU15_AD2_50		
AS01_AD2_0			AS01_AD2_15				AS01_AD2_35				AS01_AD2_55	
AS02_AD2_0				AS02_AD2_20				AS02_AD2_40				AS02_AD2_60
AS03_AD2_0	AS03_AD2_5				AS03_AD2_25				AS03_AD2_45			
AS04_AD2_0		AS04_AD2_10				AS04_AD2_30				AS04_AD2_50		
AS05_AD2_0			AS05_AD2_15				AS05_AD2_35				AS05_AD2_55	
AS06_AD2_0				AS06_AD2_20				AS06_AD2_40				AS06_AD2_60
AS07_AD2_0	AS07_AD2_5				AS07_AD2_25				AS07_AD2_45			
AS08_AD2_0		AS08_AD2_10				AS08_AD2_30				AS08_AD2_50		
AS09_AD2_0			AS09_AD2_15				AS09_AD2_35				AS09_AD2_55	
AS10_AD2_0				AS10_AD2_20				AS10_AD2_40				AS10_AD2_60
AS11_AD2_0	AS11_AD2_5				AS11_AD2_25				AS11_AD2_45			
AS12_AD2_0		AS12_AD2_10				AS12_AD2_30				AS12_AD2_50		
AS13_AD2_0			AS13_AD2_15				AS13_AD2_35				AS13_AD2_55	
AS14_AD2_0				AS14_AD2_20				AS14_AD2_40				AS14_AD2_60
AS15_AD2_0	AS15_AD2_5				AS15_AD2_25				AS15_AD2_45			
ST01_AD2_0		ST01_AD2_10				ST01_AD2_30				ST01_AD2_50		
ST02_AD2_0			ST02_AD2_15				ST02_AD2_35				ST02_AD2_55	
ST03_AD2_0				ST03_AD2_20				ST03_AD2_40				ST03_AD2_60
ST04_AD2_0	ST04_AD2_5				ST04_AD2_25				ST04_AD2_45			
ST05_AD2_0		ST05_AD2_10				ST05_AD2_30				ST05_AD2_50		
ST06_AD2_0			ST06_AD2_15				ST06_AD2_35				ST06_AD2_55	
ST07_AD2_0				ST07_AD2_20				ST07_AD2_40				ST07_AD2_60
ST08_AD2_0	ST08_AD2_5				ST08_AD2_25				ST08_AD2_45			
ST09_AD2_0		ST09_AD2_10				ST09_AD2_30				ST09_AD2_50		
ST10_AD2_0			ST10_AD2_15				ST10_AD2_35				ST10_AD2_55	
ST11_AD2_0				ST11_AD2_20				ST11_AD2_40				ST11_AD2_60
ST12_AD2_0	ST12_AD2_5				ST12_AD2_25				ST12_AD2_45			
ST13_AD2_0		ST13_AD2_10				ST13_AD2_30				ST13_AD2_50		
ST14_AD2_0			ST14_AD2_15				ST14_AD2_35				ST14_AD2_55	
ST15_AD2_0				ST15_AD2_20				ST15_AD2_40				ST15_AD2_60

Table reports the case of AD2 as example. AS = Asphodel; EU = Eucalyptus; MF = Multifloral; RO = Rosemary; ST = Strawberry tree; TH = Thistle; AD2 = second adulterant

Table S4. Division of the honey dataset into the different categories according to traceability.

**Geographical origin**			
Category	Calibration	Validation	**Total**
IT	116	52	168
SP	47	24	71
**Total**	163	76	239
**Botanical origin**			
Category	Calibration	Validation	**Total**
AS	24	12	36
EU	28	14	42
MF	45	23	68
RO	20	11	31
ST	18	7	25
TH	25	12	37
**Total**	160	79	239
**Geographical and botanical origin**			
Category	Calibration	Validation	**Total**
IT-AS	24	12	36
IT-EU	20	10	30
IT-MF	23	12	35
IT-RO	4	1	5
IT-ST	16	9	25
IT-TH	25	12	37
SP-EU	8	4	12
SP-MF	22	11	33
SP-RO	17	9	26
**Total**	159	80	239

IT = Sardinia (Italy); SP = Spain; AS = Asphodel; EU = Eucalyptus; MF = Multifloral; RO = Rosemary; ST = Strawberry tree; TH = Thistle.

Table S5. Discrimination of honeys based on geographical origin using LDA.

Spectral treatment	n. selected variables	Selected wavelengths	Percentage of correct classification	
			Cross Validation	Prediction
None	30	914.3 to 970.0 nm, 1193.0 to 1217.8 nm, 1236.4 to 1267.4 nm, 1304.5 to 1316.9 nm, 1589.5 to 1608.1 nm, 1663.8 to 1670.0 nm	96	95
1st derivative	22	926.7 to 957.7 nm, 1186.8 to 1193.0 nm, 1205.4 to 1217.8 nm, 1236.4 to 1261.2 nm, 1409.8 to 1422.2 nm, 1632.8 to 1645.2 nm	97	92
2nd derivative	23	920.5 to 963.8 nm, 1050.6 to 1056.8 nm, 1155.9 nm, 1199.2 to 1211.6 nm, 1236.4 to 1248.8 nm, 1323.1 to 1335.5 nm, 1626.6 to 1639.0 nm	97	94
SNV	15	920.5 to 945.3 nm, 1199.2 to 1217.8 nm, 1242.6 to 1273.6 nm	91	86
SNV + 1st derivative	25	926.7 to 957.7 nm, 1162.1 nm, 1186.8 to 1193.0 nm, 1205.4 to 1217.8 nm, 1236.4 to 1248.8 nm, 1261.2 nm, 1286.0 to 1298.3 nm, 1335.5 to 1341.7 nm, 1416.0 nm, 1632.8 to 1645.2 nm	97	92
SNV + 2nd derivative	44	914.3 to 982.4 nm, 1050.6 to 1069.2 nm, 1118.7 nm, 1155.9 to 1168.3 nm, 1199.2 to 1224.0 nm, 1236.4 to 1248.8 nm, 1279.8 nm, 1304.5 to 1341.7 nm, 1546.1 to 1552.3 nm, 1626.6 to 1657.6 nm	95	90
MSC	29	914.3 to 945.3 nm, 963.8 nm, 1025.8 nm, 1149.7 nm, 1199.2 to 1224.0 nm, 1236.4 to 1267.4 nm, 1310.7 to 1323.1 nm, 1403.6 to 1409.8 nm, 1595.7 to 1601.9 nm, 1663.8 to 1670.0 nm	94	93
MSC + 1st derivative	20	926.7 to 957.7 nm, 1205.4 to 1217.8 nm, 1236.4 to 1248.8 nm, 1261.2 nm, 1335.5 to 1341.7 nm, 1471.8 nm, 1552.3 nm, 1632.8 to 1645.2 nm	94	93
MSC + 2nd derivative	51	914.3 to 976.2 nm, 1050.6 to 1063.0 nm, 1112.5 to 1118.7 nm, 1143.5 to 1168.3 nm, 1180.7 to 1224.0 nm, 1236.4 to 1248.8 nm, 1279.8 to 1286.0 nm, 1304.5 to 1354.1 nm, 1546.1 nm, 1626.6 to 1657.6 nm	96	94

The common spectral regions are colored. SNV = Standard Normal Variate; MSC = multiplicative Scatter Correction,

Table S6. Discrimination of honeys based on botanical origin using LDA.

Spectral treatment	n. selected variables	Selected wavelengths	Percentage of correct classification	
			Cross Validation	Prediction
None	49	914.3 to 957.7 nm, 1001.0 to 1013.4 nm, 1069.2 to 1100.1 nm, 1292.2 to 1304.5 nm, 1329.3 to 1347.9 nm, 1360.3 nm, 1391.3 to 1403.6 nm, 1416.0 to 1428.4 nm, **1484.2 to 1496.6** nm, 1515.1 to 1521.3 nm, 1552.3 nm, 1601.9 to 1670.0 nm	85	91
1st derivative	46	926.7 to 945.3 nm, 994.8 to 1007.2 nm, 1056.8 to 1063.0 nm, 1081.5 to 1100.1 nm, 1118.7 to 1137.3 nm, 1180.7 to 1193.0 nm, 1211.6 to 1224.0 nm, 1248.8 nm, 1354.1 nm, 1397.5 nm, 1416.0 to 1422.2 nm, 1478.0 to 1496.6 nm, 1546.1 nm, 1583.3 nm, 1595.7 to 1663.8 nm	84	81
2nd derivative	41	932.9 to 951.5 nm, 1050.6 to 1063.0 nm, 1081.5 to 1087.7 nm, 1100.1 to 1124.9 nm, 1347.9 to 1366.5 nm, 1391.3 to 1416.0 nm, 1478.0 to 1509.0 nm, 1539.9 to 1546.1 nm, **1601.9 to 1657.6** nm	86	82
SNV	54	914.3 to 951.5 nm, 1007.2 to 1019.6 nm, 1075.3 to 1093.9 nm, 1106.3 to 1131.1 nm, 1155.9 to 1168.3 nm, 1199.2 nm, 1298.3 nm, 1323.1 to 1360.3 nm, 1391.3 nm, 1484.2 to 1521.3 nm, 1552.3 nm, 1583.3 nm, 1595.7 to 1670.0 nm	84	88
SNV + 1st derivative	43	**926.7 to 945.3 nm**, 994.8 to **1007.2** nm, 1063.0 nm, 1087.7 to 1100.1 nm, 1124.9 to 1137.3 nm, 1186.8 to 1193.0 nm, 1205.4 to 1224.0 nm, 1354.1 to 1360.3 nm, 1403.6 to 1416.0 nm, 1478.0 to 1490.4 nm, 1502.8 to 1509.0 nm, 1539.9 to 1546.1 nm, 1601.9 to 1663.8 nm	85	84
SNV + 2nd derivative	49	926.7 to 951.5 nm, 1032.0 nm, 1050.6 to 1087.7 nm, 1100.1 nm, 1112.5 nm, 1186.8 nm, 1248.8 nm, 1323.1 nm, 1354.1 nm, 1385.1 to 1428.4 nm, 1465.6 to 1509.0 nm, 1539.9 to 1546.1 nm, 1589.5 to 1657.6 nm	81	81
MSC	56	920.5 to 945.3 nm, 1001.0 to 1013.4 nm, 1069.2 to 1100.1 nm, 1112.5 to 1131.1 nm, 1143.5 to 1174.5 nm, 1186.8 to 1205.4 nm, 1316.9 to 1354.1 nm, 1391.3 nm, 1484.2 to 1521.3 nm, 1595.7 to 1670.0 nm	83	80
MSC + 1st derivative	50	926.7 to 945.3 nm, 994.8 to 1007.2 nm, 1056.8 to 1063.0 nm, 1081.5 to 1100.1 nm, 1124.9 to 1137.3 nm, 1186.8 to 1193.0 nm, 1205.4 to 1224.0 nm, 1354.1 to 1366.5 nm, 1409.8 to 1428.4 nm, 1478.0 to 1509.0 nm, 1533.7 to 1546.1 nm, 1589.5 nm, 1601.9 to 1663.8 nm	84	85
MSC + 2nd derivative	55	920.5 to 951.5 nm, 1050.6 to 1112.5 nm, 1124.9 nm, 1186.8 nm, 1211.6 nm, 1323.1 to 1329.3 nm, 1347.9 to 1354.1 nm, 1385.1 to 1428.4 nm, 1465.6 to 1509.0 nm, 1539.9 to 1546.1 nm, 1589.5 to 1657.6 nm	82	86

The common spectral regions are colored. SNV = Standard Normal Variate; MSC = multiplicative Scatter Correction

Table S7. Discrimination of honeys based on geographical-botanical origin using LDA.

Spectral treatment	n. selected variables	Selected wavelengths	Percentage of correct classification	
			Cross Validation	Prediction
None	50	914.3 to 939.1 nm, 970.0 to 976.2 nm, 1038.2 to 1143.5 nm, 1205.4 to 1217.8 nm, 1255.0 nm, 1471.8 to 1490.4 nm, 1509.0 to 1521.3 nm, 1546.1 to 1558.5 nm, 1595.7 to 1639.0 nm, 1657.6 to 1670.0 nm	83	85
1st derivative	50	926.7 to 957.7 nm, 1056.8 to 1063.0 nm, 1087.7 to 1162.1 nm, 1205.4 to 1217.8 nm, 1236.4 to 1248.8 nm, 1316.9 nm, 1329.3 nm, 1409.8 to 1416.0 nm, 1484.2 nm, 1502.8 to 1509.0 nm, 1546.1 to 1552.3 nm, 1583.3 to 1663.8 nm	82	81
2nd derivative	50	920.5 to 926.7 nm, 963.8 nm, 1044.4 to 1063.0 nm, 1081.5 to 1124.9 nm, 1199.2 to 1211.6 nm, 1248.8 to 1255.0 nm, 1304.5 to 1323.1 nm, 1341.7 to 1360.3 nm, 1416.0 nm, 1490.4 to 1509.0 nm, 1539.9 to 1552.3 nm, 1577.1 to 1657.6 nm	81	78
SNV	40	914.3 to 939.1 nm, 1056.8 nm, 1069.2 to 1149.7 nm, 1199.2 to 1217.8 nm, 1316.9 to 1335.5 nm, 1484.2 nm, 1552.3 to 1558.5 nm, 1595.7 to 1614.3 nm, 1632.8 to 1639.0 nm, 1657.6 to 1670.0 nm	83	83
SNV + 1st derivative	54	920.5 to 976.2 nm, 1056.8 to 1063.0 nm, 1087.7 to 1137.3 nm, 1205.4 to 1217.8 nm, 1236.4 nm, 1261.2 to 1267.4 nm, 1391.3 to 1416.0 nm, 1484.2 to 1509.0 nm, 1539.9 to 1552.3 nm, 1583.3 to 1663.8 nm	79	78
SNV + 2nd derivative	47	920.5 to 932.9 nm, 963.8 nm, 1044.4 to 1063.0 nm, 1081.5 nm, 1093.9 to 1118.7 nm, 1199.2 to 1217.8 nm, 1242.6 to 1255.0 nm, 1329.3 nm, 1341.7 to 1354.1 nm, 1403.6 to 1416.0 nm, 1490.4 to 1509.0 nm, 1546.1 to 1552.3 nm, 1583.3 to 1657.6 nm	81	81
MSC	51	920.5 to 945.3 nm, 1001.0 to 1013.4 nm, 1069.2 to 1093.9 nm, 1143.5 to 1162.1 nm, 1193.0 to 1211.6 nm, 1298.3 nm, 1316.9 to 1360.3 nm, 1416.0 to 1428.4 nm, 1484.2 to 1502.8 nm, 1515.1 nm, 1595.7 to 1670.0 nm	79	86
MSC + 1st derivative	42	920.5 to 945.3 nm, 994.8 to 1007.2 nm, 1063.0 nm, 1087.7 to 1100.1 nm, 1118.7 to 1131.1 nm, 1186.8 to 1193.0 nm, 1211.6 to 1224.0 nm,1397.5 to 1422.2 nm, 1478.0 to 1509.0 nm, 1546.1 nm, 1608.1 to 1663.8 nm	83	84
MSC + 2nd derivative	36	920.5 to 951.5 nm, 1050.6 to 1056.8 nm, 1081.5 to 1087.7 nm, 1186.8 nm, 1248.8 nm, 1329.3 nm, 1354.1 nm, 1385.1 nm, 1409.8 to 1422.2 nm, 1490.4 to 1509.0 nm, 1546.1 to 1552.3 nm, 1589.5 to 1657.6 nm	81	79

The common spectral regions are colored. SNV = Standard Normal Variate; MSC = multiplicative Scatter Correction

Table S8. Classification parameters for detecting AD1 adulteration in multifloral honeys using LDA with varying spectral pre-treatments.

Spectral pre-treatment	n. of variables	Selected wavelengths	Percentage of correct classification	
			Cross Validation	Prediction
None	25	1007.2 to 1025.8 nm, 1118.7 to 1124.9 nm, 1211.6 to 1236.4 nm, 1279.8 to 1304.5 nm, 1329.3 to 1378.9 nm	95	94
1st derivative	27	1013.4 to 1025.8 nm, 1087.7 to 1093.9 nm, 1112.5 to 1124.9 nm, 1186.8 to 1199.2 nm, 1230.2 to 1236.4 nm, 1261.2 nm, 1298.3 to 1304.5 nm, 1323.1 to 1366.5 nm, 1620.5 to 1632.8 nm	96	91
2nd derivative	45	1007.2 to 1025.8 nm, 1038.2 nm, 1081.5 to 1124.9 nm, 1143.5 to 1149.7 nm, 1211.6 to 1242.6 nm, 1298.3 to 1366.5 nm, 1403.6 nm, 1502.8 to 1515.1 nm, 1564.7 nm, 1620.5 to 1657.6 nm	96	96
SNV	34	1007.2 to 1025.8 nm, 1118.7 to 1131.1 nm, 1168.3 nm, 1193.0 to 1236.4 nm, 1255.0 nm, 1279.8 to 1378.9 nm	96	94
SNV + 1st derivative	46	1007.2 to 1044.4 nm, 1087.7 to 1100.1 nm, 1112.5 to 1131.1 nm, 1162.1 to 1199.2 nm, 1224.0 to 1279.8 nm, 1298.3 to 1304.5 nm, 1329.3 to 1360.3 nm, 1471.8 nm, 1620.5 to 1632.8 nm, 1645.2 to 1657.6 nm	97	95
SNV + 2nd derivative	50	908.1 to 914.3 nm, 988.6 to 1025.8 nm, 1069.2 to 1124.9 nm, 1149.7 to 1162.1 nm, 1199.2 to 1242.6 nm, 1298.3 nm, 1316.9 to 1372.7 nm, 1422.2 nm, 1465.6 nm, 1620.5 to 1657.6 nm	93	98
MSC	37	1007.2 to 1025.8 nm, 1081.5 to 1087.7 nm, 1118.7 to 1131.1 nm, 1186.8 to 1236.4 nm, 1267.4 to 1378.9 nm	97	95
MSC + 1st derivative	42	1013.4 to 1025.8 nm, 1087.7 to 1137.3 nm, 1162.1 to 1205.4 nm, 1224.0 to 1236.4 nm, 1255.0 to 1273.6 nm, 1292.2 to 1304.5 nm, 1329.3 to 1360.3 nm, 1620.5 to 1651.4 nm	95	96
MSC + 2nd derivative	60	908.1 to 914.3 nm, 1001.0 to 1025.8 nm, 1044.4 nm, 1069.2 to 1124.9 nm, 1149.7 to 1168.3 nm, 1193.0 to 1255.0 nm, 1298.3 to 1366.5 nm, 1391.3 to 1403.6 nm, 1459.4 to 1471.8 nm, 1558.5 to 1564.7 nm, 1620.5 to 1657.6 nm	97	100

The common spectral regions are colored. SNV = Standard Normal Variate; MSC = multiplicative Scatter Correction

Table S9. Classification parameters for detecting AD2 adulteration in multifloral honeys using LDA with varying spectral pre-treatments.

Spectral pre-treatment	n. of variables	Selected wavelengths	Percentage of correct classification	
			Cross Validation	Prediction
None	38	939.1 to 945.3 nm, 1162.1 to 1286.0 nm, 1341.7 to 1391.3 nm, 1509.0 to 1515.1 nm, 1577.1 to 1583.3 nm, 1651.4 to 1657.6 nm	90	96
1st derivative	33	932.9 to 945.3 nm, 1124.9 to 1137.3 nm, 1155.9 to 1211.6 nm, 1236.4 to 1255.0 nm, 1341.7 to 1354.1 nm, 1515.1 to 1521.3 nm, 1570.9 to 1583.3 nm, 1614.3 to 1632.8 nm, 1663.8 nm	91	95
2nd derivative	52	914.3 to 932.9 nm, 1112.5 to 1180.7 nm, 1193.0 to 1224.0 nm, 1255.0 nm, 1304.5 to 1329.3 nm, 1347.9 nm, 1360.3 to 1422.2 nm, 1509.0 to 1515.1 nm, 1564.7 to 1583.3 nm, 1608.1 to 1639.0 nm	92	98
SNV	52	939.1 to 951.5 nm, 1131.1 nm, 1143.5 to 1335.5 nm, 1366.5 to 1391.3 nm, 1502.8 to 1509.0 nm, 1570.9 to 1595.7 nm, 1645.2 to 1663.8 nm	92	96
SNV + 1st derivative	51	914.3 nm, 932.9 to 945.3 nm, 1038.2 to 1044.4 nm, 1124.9 to 1211.6 nm, 1224.0 to 1261.2 nm, 1304.5 to 1316.9 nm, 1341.7 to 1354.1 nm, 1372.7 to 1385.1 nm, 1502.8 to 1515.1 nm, 1570.9 to 1583.3 nm, 1614.3 to 1645.2 nm, 1663.8 to 1670.0 nm	92	99
SNV + 2nd derivative	51	914.3 to 932.9 nm, 988.6 nm, 1106.3 to 1180.7 nm, 1193.0 to 1230.2 nm, 1248.8 to 1255.0 nm, 1316.9 to 1329.3 nm, 1385.1 to 1409.8 nm, 1422.2 to 1428.4 nm, 1496.6 to 1515.1 nm, 1564.7 to 1583.3 nm, 1608.1 to 1639.0 nm	90	99
MSC	63	914.3 to 945.3 nm, 1124.9 to 1397.5 nm, 1502.8 to 1515.1 nm, 1570.9 to 1595.7 nm, 1645.2 to 1663.8 nm	93	98
MSC + 1st derivative	35	914.3 to 945.3 nm, 1106.3 to 1242.6 nm, 1329.3 to 1341.7 nm, 1570.9 to 1583.3 nm	92	99
MSC + 2nd derivative	58	914.3 to 932.9 nm, 1100.1 to 1224.0 nm, 1316.9 to 1329.3 nm, 1360.3 to 1409.8 nm, 1422.2 to 1434.6 nm, 1490.4 to 1515.1 nm, 1564.7 to 1583.3 nm, 1608.1 to 1657.6 nm	93	96

The common spectral regions are colored. SNV = Standard Normal Variate; MSC = multiplicative Scatter Correction

Table S10. Classification parameters for detecting AD1 adulteration in unifloral honeys using LDA with varying spectral pre-treatments.

Spectral pre-treatment	n. of variables	Selected wavelengths	Percentage of correct classification	
			Cross Validation	Prediction
None	46	914.3 to 932.9 nm, 951.5 to 957.7 nm, 1013.4 nm, 1056.8 to 1069.2 nm, 1118.7 to 1137.3 nm, 1211.6 to 1236.4 nm, 1255.0 to 1391.3 nm, 1552.3 nm, 1608.1 to 1620.5 nm	94	97
1st derivative	41	939.1 to 951.5 nm, 1118.7 to 1124.9 nm, 1174.5 to 1193.0 nm, 1230.2 to 1242.6 nm, 1292.2 to 1304.5 nm, 1329.3 to 1409.8 nm, 1478.0 to 1484.2 nm, 1539.9 to 1552.3 nm, 1601.9 to 1614.3 nm, 1626.6 to 1645.2 nm	95	95
2nd derivative	27	1199.2 to 1205.4 nm, 1323.1 to 1391.3 nm, 1409.8 to 1422.2 nm, 1533.7 to 1552.3 nm, 1620.5 to 1651.4 nm	96	91
SNV	55	914.3 to 932.9 nm, 951.5 to 957.7 nm, 1013.4 to 1025.8 nm, 1056.8 to 1069.2 nm, 1112.5 to 1137.3 nm, 1205.4 to 1242.6 nm, 1255.0 to 1341.7 nm, 1354.1 to 1378.9 nm, 1539.9 to 1552.3 nm, 1608.1 to 1651.4 nm	95	96
SNV + 1st derivative	27	939.1 to 951.5 nm, 1112.5 to 1124.9 nm, 1224.0 to 1255.0 nm, 1335.5 to 1360.3 nm, 1478.0 nm, 1539.9 to 1552.3 nm, 1601.9 to 1614.3 nm, 1632.8 to 1645.2 nm	96	96
SNV + 2nd derivative	35	920.5 to 926.7 nm, 1106.3 to 1118.7 nm, 1199.2 to 1205.4 nm, 1323.1 to 1385.1 nm, 1409.8 to 1422.2 nm, 1533.7 to 1552.3 nm, 1601.9 to 1657.6 nm	95	91
MSC	49	914.3 to 932.9 nm, 945.3 to 957.7 nm, 1013.4 to 1025.8 nm, 1056.8 to 1069.2 nm, 1124.9 to 1137.3 nm, 1211.6 to 1236.4 nm, 1255.0 to 1385.1 nm, 1546.1 to 1558.5 nm, 1608.1 to 1620.5 nm	96	97
MSC + 1st derivative	33	920.5 to 926.7 nm, 939.1 to 951.5 nm, 1112.5 to 1124.9 nm, 1174.5 to 1186.8 nm, 1224.0 to 1255.0 nm, 1335.5 to 1360.3 nm, 1539.9 to 1558.5 nm, 1601.9 to 1614.3 nm, 1626.6 to 1645.2 nm	96	95
MSC + 2nd derivative	47	920.5 to 926.7 nm, 1106.3 to 1137.3 nm, 1168.3 to 1174.5 nm, 1199.2 to 1211.6 nm, 1286.0 to 1298.3 nm, 1323.1 to 1422.2 nm, 1527.5 to 1552.3 nm, 1601.9 nm, 1614.3 to 1657.6 nm	96	91

The common spectral regions are colored. SNV = Standard Normal Variate; MSC = multiplicative Scatter Correction

Table S11. Classification parameters for detecting AD2 adulteration in unifloral honeys using LDA with varying spectral pre-treatments.

Spectral pre-treatment	n. of variables	Selected wavelengths	Percentage of correct classification	
			Cross Validation	Prediction
None	52	920.5 to 945.3 nm, 970.0 to 982.4 nm, 1069.2 to 1112.5 nm, 1143.5 to 1180.7 nm, 1224.0 to 1230.2 nm, 1279.8 to 1347.9 nm, 1385.1 to 1403.6 nm, 1601.9 to 1608.1 nm, 1620.5 to 1670.0 nm	97	100
1st derivative	55	914.3 to 951.5 nm, 976.2 nm, 1069.2 to 1075.3 nm, 1093.9 to 1106.3 nm, 1149.7 to 1224.0 nm, 1267.4 to 1310.7 nm, 1335.5 to 1403.6 nm, 1515.1 to 1527.5 nm, 1608.1 to 1614.3 nm, 1626.6 to 1645.2 nm	97	99
2nd derivative	34	920.5 to 932.9 nm, 1149.7 to 1174.5 nm, 1211.6 to 1224.0 nm, 1273.6 to 1286.0 nm, 1304.5 to 1366.5 nm, 1589.5 to 1595.7 nm, 1608.1 to 1645.2 nm	96	96
SNV	21	926.7 to 939.1 nm, 970.0 to 982.4 nm, 1013.4 nm, 1155.9 to 1180.7 nm, 1217.8 to 1230.2 nm, 1378.9 to 1409.8 nm	96	100
SNV + 1st derivative	26	1001.0 to 1013.4 nm, 1100.1 to 1106.3 nm, 1155.9 to 1217.8 nm, 1279.8 to 1292.2 nm, 1316.9 to 1323.1 nm, 1347.9 nm, 1632.8 to 1651.4 nm	97	100
SNV + 2nd derivative	51	920.5 to 932.9 nm, 945.3 nm, 1007.2 nm, 1093.9 to 1106.3 nm, 1143.5 to 1174.5 nm, 1205.4 to 1224.0 nm, 1261.2 to 1286.0 nm, 1298.3 to 1372.7 nm, 1385.1 to 1397.5 nm, 1583.3 to 1589.5 nm, 1601.9 to 1657.6 nm	96	96
MSC	22	926.7 to 939.1 nm, 970.0 to 982.4 nm, 1013.4 nm, 1100.1 to 1112.5 nm, 1162.1 to 1180.7 nm, 1217.8 to 1230.2 nm, 1378.9 to 1403.6 nm	97	99
MSC + 1st derivative	56	926.7 nm, 939.1 to 957.7 nm, 976.2 nm, 1001.0 to 1013.4 nm, 1075.3 to 1106.3 nm, 1149.7 to 1224.0 nm, 1286.0 to 1347.9 nm, 1372.7 to 1409.8 nm, 1608.1 to 1663.8 nm	97	100
MSC + 2nd derivative	42	920.5 to 932.9 nm, 1050.6 nm, 1100.1 to 1106.3 nm, 1143.5 to 1174.5 nm, 1211.6 to 1224.0 nm, 1273.6 to 1286.0 nm, 1298.3 to 1341.7 nm, 1360.3 to 1397.5 nm, 1608.1 to 1657.6 nm	97	97

The common spectral regions are colored. SNV = Standard Normal Variate; MSC = multiplicative Scatter Correction

Table S12. Prediction parameters for detecting AD1 adulteration in honeys using PLS with varying spectral pre-treatments.

Spectral pre-treatment	Variable selection	n. of variables	Selected variables (wavelengths)	LV	Explained Variance (%)	Calibration			Validation			
						Median	RMSECV	RMSECV%	Median	RMSEP	RMSEP%	bias
None	None	125	whole spectrum	10	95.16	0.315	0.045	14.3	0.34	0.04	11.8	0.012
	GA	30	970.0 nm, 988.6 to 1007.2 nm, 1168.3 to 1186.8 nm, 1211.6 to 1236.4 nm, 1273.6 to 1378.9 nm	10	96.22	0.31	0.04	12.9	0.340	0.045	13.2	0.010
1st derivative	None	125	whole spectrum	14	92.80	0.315	0.055	17.5	0.34	0.05	14.7	0.005
	GA	44	982.4 nm, 1124.9 to 1149.7 nm, 1168.3 to 1205.4 nm, 1224.0 to 1316.9 nm, 1329.3 to 1397.5 nm, 1614.3 to 1626.6 nm	10	88.04	0.31	0.07	22.6	0.340	0.065	19.1	0.005
2nd derivative	None	125	whole spectrum	10	90.03	0.315	0.065	20.6	0.34	0.06	17.6	-0.0004
	GA	40	988.6 nm, 1149.7 to 1155.9 nm, 1193.0 to 1248.8 nm, 1286.0 to 1304.5 nm, 1329.3 to 1366.5 nm, 1626.6 to 1645.2 nm	14	93.57	0.31	0.05	16.1	0.34	0.06	17.6	0.0075
SNV	None	125	whole spectrum	9	95.31	0.315	0.045	14.3	0.34	0.04	11.8	0.010
	GA	14	994.8 to 1007.2 nm, 1168.3 nm, 1217.8 to 1230.2 nm, 1279.8 nm, 1298.3 to 1304.5 nm, 1323.1 to 1329.3 nm, 1360.3 to 1366.5 nm	10	95.86	0.31	0.04	12.9	0.34	0.04	11.8	0.009
SNV + 1st derivative	None	125	whole spectrum	6	94.93	0.31	0.05	16.1	0.34	0.04	11.8	0.007
	GA	21	1131.1 nm, 1174.5 to 1205.4 nm, 1224.0 to 1267.4 nm, 1292.2 to 1298.3 nm, 1335.5 to 1354.1 nm	8	95.20	0.315	0.045	14.3	0.34	0.05	14.7	0.008
SNV + 2nd derivative	None	125	whole spectrum	13	91.09	0.31	0.06	19.4	0.34	0.05	14.7	0.001
	GA	34	988.6 nm, 1106.3 to 1112.5 nm, 1124.9 nm, 1137.3 to 1174.5 nm, 1199.2 to 1230.2 nm, 1286.0 to 1310.7 nm, 1323.1 to 1366.5 nm, 1626.6 to 1645.2 nm	15	93.38	0.31	0.05	16.1	0.34	0.05	14.7	0.0065
MSC	None	125	whole spectrum	10	95.41	0.31	0.04	12.9	0.34	0.04	11.8	0.009
	GA	13	994.8 to 1001.0 nm, 1168.3 nm, 1217.8 to 1230.2 nm, 1292.1 to 1304.5 nm, 1323.1 to 1329.3 nm, 1354.1 to 1360.3 nm	9	95.83	0.31	0.04	12.9	0.34	0.04	11.8	0.006
MSC + 1st derivative	None	125	whole spectrum	6	94.93	0.31	0.05	16.1	0.34	0.04	11.8	0.007
	GA	26	1131.1 to 1137.3 nm, 1174.5 to 1205.4 nm, 1224.0 to 1273.6 nm, 1292.2 to 1304.5 nm, 1335.5 to 1360.3 nm, 1372.7 nm	6	95.02	0.31	0.05	16.1	0.34	0.05	14.7	0.009
MSC + 2nd derivative	None	125	whole spectrum	13	91.08	0.31	0.06	19.4	0.34	0.05	14.7	0.001
	GA	32	1112.5 nm, 1149.7 to 1174.5 nm, 1193.0 to 1242.6 nm, 1286.0 to 1310.7 nm, 1323.1 to 1366.5 nm, 1626.6 to 1645.2 nm	15	93.35	0.31	0.05	16.1	0.34	0.05	14.7	0.007

The common spectral regions are colored. SNV = Standard Normal Variate; MSC = multiplicative Scatter Correction; GA = Genetic Algorithms; LV = Latent Variables; RMSECV = Root Mean Square Error in Cross Validation; RMSEP = Root Mean Square Error in Prediction.

Table S13. Prediction parameters for detecting AD2 adulteration in multifloral honeys using PLS with varying spectral pre-treatments.

Spectral pre-treatment	Variable selection	n. of variables	Selected variables (wavelengths)	LV	Explained Variance (%)	Calibration			Validation			
						Median	RMSECV	RMSECV%	Median	RMSEP	RMSEP%	bias
None	None	125	whole spectrum	13	90.35	0.30	0.06	20.0	0.31	0.07	22.6	-0.0064
	GA	36	988.6 to 1007.2 nm, 1056.8 to 1063.0 nm, 1162.1 to 1199.2 nm, 1211.6 to 1267.4 nm, 1316.9 to 1391.3 nm	15	88.79	0.30	0.07	23.3	0.31	0.07	22.6	-0.0087
1st derivative	None	125	whole spectrum	10	90.33	0.30	0.06	20.0	0.31	0.07	22.6	-0.0019
	GA	20	1174.5 to 1205.4 nm, 1230.2 to 1255.0 nm, 1341.7 to 1360.3 nm, 1391.3 to 1416.0 nm	10	89.69	0.30	0.07	23.3	0.31	0.08	25.8	-0.0067
2nd derivative	None	125	whole spectrum	7	83.51	0.30	0.08	26.7	0.31	0.08	25.8	-0.0012
	GA	32	1131.1 to 1143.5 nm, 1162.1 to 1230.2 nm, 1242.6 to 1255.0 nm, 1316.9 to 1347.9 nm, 1397.5 to 1422.2 nm, 1620.5 to 1632.8 nm	10	88.78	0.30	0.07	23.3	0.31	0.08	25.8	-0.0092
SNV	None	125	whole spectrum	13	91.35	0.30	0.06	20.0	0.31	0.07	22.6	-0.0037
	GA	34	982.4 to 994.8 nm, 1155.9 to 1193.0 nm, 1205.4 to 1273.6 nm, 1316.9 to 1385.1 nm	8	89.22	0.30	0.07	23.3	0.31	0.07	22.6	-0.0043
SNV + 1st derivative	None	125	whole spectrum	9	91.58	0.30	0.06	20.0	0.31	0.07	22.6	-0.0069
	GA	36	1118.7 to 1162.1 nm, 1174.5 to 1211.6 nm, 1224.0 to 1255.0 nm, 1335.5 to 1360.3 nm, 1385.1 to 1416.0 nm, 1608.1 to 1614.3 nm, 1639.0 to 1645.2 nm	9	89.90	0.305	0.065	21.3	0.31	0.07	22.6	-0.0089
SNV + 2nd derivative	None	125	whole spectrum	11	85.10	0.30	0.08	26.7	0.31	0.07	22.6	-0.0105
	GA	42	1137.3 to 1143.5 nm, 1155.9 to 1255.0 nm, 1316.9 to 1329.3 nm, 1360.3 to 1366.5 nm, 1391.3 to 1422.2 nm, 1471.8 to 1484.2 nm, 1608.1 nm, 1620.5 to 1663.8 nm	8	89.15	0.30	0.07	23.3	0.31	0.08	25.8	-0.0108
MSC	None	125	whole spectrum	12	90.72	0.30	0.06	20.0	0.31	0.07	22.6	-0.0068
	GA	35	982.4 to 994.8 nm, 1056.8 nm, 1137.3 nm, 1162.1 to 1273.6 nm, 1316.9 to 1378.9 nm	10	90.35	0.30	0.06	20.0	0.31	0.07	22.6	-0.0039
MSC + 1st derivative	None	125	whole spectrum	9	91.55	0.30	0.06	20.0	0.31	0.07	22.6	-0.0070
	GA	21	1118.7 to 1162.1 nm, 1174.5 to 1211.6 nm, 1224.0 to 1255.0 nm	12	77.88	0.3	0.1	33.3	0.31	0.09	29.0	-0.0123
MSC + 2nd derivative	None	125	whole spectrum	11	85.13	0.30	0.08	26.7	0.31	0.07	22.6	-0.0106
	GA	40	1143.5 to 1230.2 nm, 1242.6 to 1255.0 nm, 1316.9 to 1329.3 nm 1391.3 to 1422.2 nm, 1471.8 to 1484.2 nm, 1608.1 to 1663.8 nm	15	88.35	0.30	0.07	23.3	0.31	0.08	25.8	-0.0109

The common spectral regions are colored. SNV = Standard Normal Variate; MSC = multiplicative Scatter Correction; GA = Genetic Algorithms; LV = Latent Variables; RMSECV = Root Mean Square Error in Cross Validation; RMSEP = Root Mean Square Error in Prediction.

Table S14. Prediction parameters for detecting AD1 adulteration in unifloral honeys using PLS with varying spectral pre-treatments.

Spectral pre-treatment	Variable selection	n. of variables	Selected variables (wavelengths)	LV	Explained Variance (%)	Calibration			Validation			
						Median	RMSECV	RMSECV%	Median	RMSEP	RMSEP%	bias
None	None	125		14	96.00	0.33	0.04	12.1	0.33	0.03	9.1	0.0006
	GA	28	951.5 to 957.7 nm, 988.6 to 1013.4 nm, 1162.1 to 1174.5 nm, 1211.6 to 1230.2 nm, 1267.4 to 1279.8 nm, 1304.5 to 1329.3 nm, 1360.3 to 1391.3 nm	7	96.39	0.33	0.04	12.1	0.33	0.04	12.1	0.002
1st derivative	None	125		10	97.50	0.33	0.03	9.1	0.33	0.03	9.1	0.004
	GA	25	1168.3 to 1199.2 nm, 1224.0 to 1255.0 nm, 1292.2 to 1304.5 nm, 1329.3 to 1385.1 nm	8	95.10	0.33	0.045	13.6	0.33	0.04	12.1	0.004
2nd derivative	None	125		13	92.54	0.33	0.06	18.2	0.33	0.05	15.2	0.007
	GA	32	914.3 to 920.5 nm, 1069.2 to 1081.5 nm, 1112.5 o 1118.7 nm, 1162.1 to 1174.5 nm, 1193.0 to 1217.8 nm, 1286.0 to 1304.5 nm, 1329.3 nm, 1347.9 to 1372.7 nm, 1484.2 to 1490.4 nm, 1632.8 to 1657.6 nm	10	93.05	0.33	0.05	15.2	0.33	0.06	18.2	0.009
SNV	None	125		8	96.27	0.33	0.04	12.1	0.33	0.04	12.1	0.003
	GA	20	945.3 to 957.7 nm, 988.6 to 994.8 nm, 1211.6 to 1230.2 nm, 1261.2 to 1273.6 nm, 1310.7 to 1329.3 nm, 1360.3 to 1372.7 nm, 1391.3 nm	10	96.16	0.33	0.04	12.1	0.33	0.04	12.1	0.006
SNV + 1st derivative	None	125		8	96.58	0.33	0.04	12.1	0.33	0.04	12.1	0.006
	GA	44	1013.4 to 1019.6 nm, 1112.5 to 1261.2 nm, 1292.2 to 1391.3 nm	7	96.08	0.33	0.04	12.1	0.33	0.04	12.1	0.003
SNV + 2nd derivative	None	125		13	93.16	0.33	0.05	15.2	0.33	0.06	18.2	0.009
	GA	25	1069.2 to 1081.5 nm, 1168.3 nm, 1199.2 to 1217.8 nm, 1286.0 to 1304.5 nm, 1323.1 to 1366.5 nm, 1632.8 to 1657.6 nm	9	92.38	0.33	0.06	18.2	0.33	0.06	18.2	0.007
MSC	None	125		8	96.27	0.33	0.04	12.1	0.33	0.04	12.1	0.002
	GA	19	945.3 to 957.7 nm, 988.6 nm, 1211.6 to 1230.2 nm, 1261.2 to 1273.6 nm, 1310.7 to 1323.1 nm, 1360.3 to 1372.7 nm, 1391.3 to 1397.5 nm	10	96.14	0.33	0.04	12.1	0.33	0.04	12.1	0.005
MSC + 1st derivative	None	125		8	96.59	0.33	0.04	12.1	0.33	0.04	12.1	0.006
	GA	38	1019.6 nm, 1112.5 to 1143.5 nm, 1168.3 to 1199.2 nm, 1217.8 to 1267.4 nm, 1286.0 to 1316.9 nm, 1329.3 to 1385.1 nm	8	95.89	0.33	0.04	12.1	0.33	0.04	12.1	0.002
MSC + 2nd derivative	None	125		13	93.15	0.33	0.05	15.2	0.33	0.06	18.2	0.009
	GA	27	1069.2 to 1081.5 nm, 1162.1 to 1168.3 nm, 1199.2 to 1217.8 nm, 1286.0 to 1310.7 nm, 1323.1 to 1366.5 nm, 1632.8 to 1657.6 nm	6	93.37	0.33	0.05	15.2	0.33	0.06	18.2	0.007

The common spectral regions are colored. SNV = Standard Normal Variate; MSC = multiplicative Scatter Correction; GA = Genetic Algorithms; LV = Latent Variables; RMSECV = Root Mean Square Error in Cross Validation; RMSEP = Root Mean Square Error in Prediction.

Table S15. Prediction parameters for detecting AD2 adulteration in unifloral honeys using PLS with varying spectral pre-treatments.

Spectral pre-treatment	Variable selection	n. of variables	Selected variables (wavelengths)	LV	Explained Variance (%)	Calibration			Validation			
						Median	RMSECV	RMSECV%	Median	RMSEP	RMSEP%	bias
None	None	125		11	92.95	0.35	0.05	14.3	0.31	0.06	19.4	-0.0002
	GA	25	745.3 to 963.8 nm, 994.8 to 1032.0 nm, 1162.1 to 1186.8 nm, 1217.8 to 1236.4 nm, 1360.3 to 1378.9 nm, 1670.0 nm	14	91.74	0.35	0.06	17.1	0.310	0.065	21.0	-0.002
1st derivative	None	125		7	89.53	0.35	0.07	20.0	0.31	0.07	22.6	0.001
	GA	13	1174.5 to 1217.8 nm, 1354.1 nm, 1539.9 to 1546.1 nm, 1663.8 to 1670.0 nm	8	91.21	0.35	0.06	17.1	0.31	0.07	22.6	-0.01
2nd derivative	None	125		7	85.48	0.35	0.08	22.9	0.31	0.07	22.6	-0.006
	GA	5	1205.4 to 1224.0 nm, 1323.1 nm	5	87.11	0.35	0.07	20.0	0.31	0.07	22.6	-0.009
SNV	None	125		12	94.00	0.35	0.05	14.3	0.31	0.05	16.1	0.003
	GA	24	945.3 to 957.7 nm, 1025.8 nm, 1162.1 to 1186.8 nm, 1211.6 to 1236.4 nm, 1360.3 to 1416.0 nm	11	94.32	0.35	0.05	14.3	0.31	0.05	16.1	-0.0003
SNV + 1st derivative	None	125		8	93.30	0.35	0.05	14.3	0.31	0.05	16.1	-0.002
	GA	28	976.2 to 982.4 nm, 1168.3 to 1224.0 nm, 1255.0 to 1267.4 nm, 1347.9 to 1385.1 nm, 1484.2 to 1490.4 nm, 1645.2 to 1663.8 nm	6	92.42	0.35	0.06	17.1	0.310	0.065	21.0	-0.004
SNV + 2nd derivative	None	125		6	86.84	0.35	0.07	20.0	0.31	0.07	22.6	-0.01
	GA	14	1162.1 to 1174.5 nm, 1205.4 to 1230.2 nm, 1329.3 nm, 1484.2 nm, 1639.0 to 1657.6 nm	15	90.51	0.35	0.06	17.1	0.31	0.09	29.0	0.06
MSC	None	125		11	93.92	0.35	0.05	14.3	0.31	0.05	16.1	0.004
	GA	21	945.3 to 957.7 nm, 1019.6 to 1025.8 nm, 1162.1 to 1186.8 nm, 1217.8 to 1230.2 nm 1366.5 to 1409.8 nm	15	94.27	0.35	0.05	14.3	0.31	0.05	16.1	0.00005
MSC + 1st derivative	None	125		8	93.28	0.35	0.05	14.3	0.31	0.05	16.1	-0.002
	GA	28	1168.3 to 1230.2 nm, 1255.0 to 1267.4 nm, 1347.9 to 1360.3 nm, 1378.9 to 1391.3 nm, 1478.0 to 1490.4 nm, 1552.3 nm, 1645.2 to 1663.8 nm	8	93.89	0.35	0.05	14.3	0.31	0.05	16.1	-0.008
MSC + 2nd derivative	None	125		6	86.85	0.35	0.07	20.0	0.31	0.07	22.6	-0.01
	GA	13	1162.1 to 1168.3 nm, 1205.4 to 1230.2 nm, 1329.3 nm, 1484.2 nm, 1639.0 to 1657.6 nm	7	90.21	0.35	0.06	17.1	0.31	0.07	22.6	-0.01

The common spectral regions are colored. SNV = Standard Normal Variate; MSC = multiplicative Scatter Correction; GA = Genetic Algorithms; LV = Latent Variables; RMSECV = Root Mean Square Error in Cross Validation; RMSEP = Root Mean Square Error in Prediction.
